# Evolutionary history of tyrosine-supplementing endosymbionts in pollen-feeding beetles

**DOI:** 10.1093/ismejo/wrae080

**Published:** 2024-06-11

**Authors:** Jürgen C Wierz, Matthew L Gimmel, Selina Huthmacher, Tobias Engl, Martin Kaltenpoth

**Affiliations:** Department of Insect Symbiosis, Max Planck Institute for Chemical Ecology, 07745 Jena, Germany; Department of Evolutionary Ecology, Institute of Organismic and Molecular Evolution, Johannes Gutenberg University, 55128 Mainz, Germany; Department of Invertebrate Zoology, Santa Barbara Museum of Natural History, Santa Barbara, CA 93105, United States; Department of Evolutionary Ecology, Institute of Organismic and Molecular Evolution, Johannes Gutenberg University, 55128 Mainz, Germany; Department of Insect Symbiosis, Max Planck Institute for Chemical Ecology, 07745 Jena, Germany; Department of Evolutionary Ecology, Institute of Organismic and Molecular Evolution, Johannes Gutenberg University, 55128 Mainz, Germany; Department of Insect Symbiosis, Max Planck Institute for Chemical Ecology, 07745 Jena, Germany; Department of Evolutionary Ecology, Institute of Organismic and Molecular Evolution, Johannes Gutenberg University, 55128 Mainz, Germany

**Keywords:** Coleoptera, Melyridae, Dasytinae, pollen feeding, *Candidatus* Dasytiphilus stammeri, bacteriome, mutualism, tyrosine supplementation, genome erosion, host symbiont coevolution, cocladogenesis

## Abstract

Many insects feeding on nutritionally challenging diets like plant sap, leaves, or wood engage in ancient associations with bacterial symbionts that supplement limiting nutrients or produce digestive or detoxifying enzymes. However, the distribution, function, and evolutionary dynamics of microbial symbionts in insects exploiting other plant tissues or relying on a predacious diet remain poorly understood. Here, we investigated the evolutionary history and function of the intracellular gamma-proteobacterial symbiont “*Candidatus* Dasytiphilus stammeri” in soft-winged flower beetles (Coleoptera, Melyridae, Dasytinae) that transition from saprophagy or carnivory to palynivory (pollen-feeding) between larval and adult stage. Reconstructing the distribution of the symbiont within the Dasytinae phylogeny unraveled not only a long-term coevolution, originating from a single acquisition event with subsequent host–symbiont codiversification, but also several independent symbiont losses. The analysis of 20 different symbiont genomes revealed that their genomes are severely eroded. However, the universally retained shikimate pathway indicates that the core metabolic contribution to their hosts is the provisioning of tyrosine for cuticle sclerotization and melanization. Despite the high degree of similarity in gene content and order across symbiont strains, the capacity to synthesize additional essential amino acids and vitamins and to recycle urea is retained in some but not all symbionts, suggesting ecological differences among host lineages. This report of tyrosine-provisioning symbionts in insects with saprophagous or carnivorous larvae and pollen-feeding adults expands our understanding of tyrosine supplementation as an important symbiont-provided benefit across a broad range of insects with diverse feeding ecologies.

## Introduction

Beetles (Coleoptera) are the most speciose of all insect orders and show a remarkable diversity in physiological and morphological traits [1]. Their defining characteristic is the modification of the anterior pair of wings into protective wing cases, called elytra, which provide efficient protection against predators and various environmental stressors such as desiccation [2, 3], allowing the colonization of diverse habitats. However, the high investment into the elytral cuticle comes at a cost, as beetles require high amounts of tyrosine during development. This aromatic amino acid is the precursor for 3,4-dihydroxyphenylalanine (DOPA), a central metabolite in the biosynthesis of melanin that serves for cuticle tanning, as well as phenolic compounds used for cuticle hardening (sclerotization) [4]. Insects in general lack the shikimate pathway for the biosynthesis of aromatic compounds and therefore are not able to produce tyrosine *de novo*. Hence, they rely on taking up aromatic amino acids via the food or acquiring them with the help of microbial symbionts.

Symbiotic interactions between insects and bacteria are widespread [5, 6], and symbiont-provided nutritional supplements enable many insect taxa to thrive in otherwise inhospitable ecological niches [7–10]. Consequently, symbiont acquisition or loss events can shape the ecology of the hosts. A new symbiont acquisition can allow a host to transition between ecological niches and compensate for a change in nutrient availability. Symbionts producing tyrosine or its precursors have been described in herbivorous beetles of the families Curculionidae, Silvanidae, and Bostrichidae [11–14], as well as in certain ants [15, 16]. Experimental deprivation of tyrosine-supplementing symbionts can result in a lighter, softer cuticle [11, 12, 17], which reduces the protection from desiccation [12, 18] as well as from predation and pathogen infestation [19].

Intimate long-term associations, including those with tyrosine-provisioning microorganisms, can have a severe impact on the symbionts’ genome evolution. As vertically transmitted symbionts permanently live in the stable environment of the host, many genes experience relaxed selection and consequently deteriorate [20]. Such genome reduction usually eliminates genes that are neither required by the symbiont nor beneficial to the host [21], whereas symbiont capabilities that are advantageous to the host are maintained [22]. As a result, the gene repertoire gets fine-tuned according to the hosts’ needs, and even in closely related coevolving symbiotic systems, the symbionts’ capabilities can differ in key elements depending on the ecology of the host [23]. A common adaptation to compensate for the loss of genes and thus reduce the effects of genome erosion is multifunctional symbiont enzymes [24]. Furthermore, hosts can sometimes compensate for symbiont gene losses, completing otherwise fragmentary pathways [25]. For example, some weevils encode a gene for the final step in the tyrosine biosynthesis pathway, i.e. tyrosine transaminase, utilizing precursors that are supplied by their *Nardonella* symbionts, which encode the shikimate pathway but lack the transaminase [11].

Soft-winged flower beetles (Melyridae) form the most speciose beetle family within the polyphagous superfamily Cleroidea, with over 6000 described species in ~300 genera and three subfamilies worldwide [26–28]. Many species may play important roles in pollination, as adults are found on flowers, often in large aggregations, and feed on pollen [29, 30]. By contrast, larvae have been recorded in leaf litter and dead wood and may be xylophagous, saprophagous, predacious, or scavenge on dead arthropods [26, 31, 32]. Moreover, it seems that larvae of some species switch from being scavengers to herbivores during their development [33]. Whereas a diet composed of arthropod prey or carcasses usually is nutrient rich, a pollen-based diet can be scarce in certain nutrients [34, 35]. Adults of both sexes from at least some species within the family carry as-yet functionally uncharacterized bacteriome-localized symbionts [9, 36]. More specifically, *Dasytes virens* and *D. plumbeus*, members of the subfamily Dasytinae, harbor a single intracellular *Enterobacteriaceae* symbiont called “*Candidatus* Dasytiphilus stammeri” (henceforth “*Dasytiphilus*”) [36]. However, no bacteriome-housed symbionts have been reported for species in other Melyridae subfamilies. Moreover, a different lifestyle has been observed in other subfamilies, where the adults can be aggressive and prey on small insects, as well as on each other [37].

Beyond its morphological and taxonomic description, little is known about this symbiotic system, which is surprising for a beetle family of this size and potential influence on ecosystem services. Hence, we investigated 58 species of Melyridae for the presence of bacterial symbionts by a combination of high-throughput 16S rRNA gene-based microbiota profiling, diagnostic PCRs, metagenome sequencing, and fluorescence *in situ* hybridization (FISH). We reconstructed a molecular phylogeny of the hosts to elucidate the origin and evolutionary dynamics of the association with nutritional symbionts, supporting a single acquisition of bacteriome-localized symbionts in Dasytinae and multiple loss events. In order to gain functional insights, we assembled the symbiont genomes of 20 host species, revealing tyrosine biosynthesis as a conserved pathway across all investigated *Dasytiphilus* strains, with additional amino acid and vitamin biosynthetic pathways being ancestrally encoded and retained in genomes of certain symbiont subclades but lost in others. These findings elucidate the function and dynamic evolutionary history of intracellular symbionts in pollen-feeding Dasytinae beetles and highlight the importance of tyrosine supplementation for the ecology and evolution across beetle families with diverse feeding ecologies.

## Material and methods

### Melyridae species collection

Melyridae beetles were collected during the summers of 2011–22 from various locations (Table 1). Individuals were stored either in ethanol or dry at −20°C or −80°C, depending on future use. Species from the USA and *Malachius bipustulatus* were identified based on morphology and species from Europe by barcoding of the CO1 gene (see below for details).

**Table 1:** List of Melyridae (and one Lophocateridae) species screened for the Dasytinae-specific symbiont *Candidatus* Dasytiphilus stammeri. Taxa are ordered by clade in the host phylogeny, separated by lines, and then alphabetically. The presence of *Dasytiphilus* was assessed by PCR, Sanger sequencing, FISH, and Illumina 16S rRNA gene amplicon profiling; empty cells indicate that this method was not used to assess symbiont presence in the respective host species. “Genome information” gives information about the results of Illumina shotgun sequencing of the symbiont genome, with length in base pairs and the GC content.

**Host**		**Collection**	**Symbiont presence confirmation method**					**Genome information**		
**Genus**	**Species**	**Country**	**Symbiont present**	**PCR**	**Sanger**	**FISH**	**Amplicon**	**Genome available**	**Genome length (bp)**	**GC%**
*Eronyxa*	*pallida*	USA, CA	No	No	No		No			
Eronyxa pallida										
*Malachius*	*bipustulatus*	Germany	No			No				
*Malachius*	*viridulus*	USA, CA	No				No			
Malachius viridulus										
*Dasytastes*	*bicolor*	USA, CA	Yes	Yes			Yes	Yes	589 892	33.0
*“Dasytes”*	*lineellus*	USA, CA	Yes	Yes	Yes		Yes	Yes	585 061	32.6
*“Dasytes”*	*seminudus*	USA, CA	Yes	Yes	Yes		Yes	Yes (draft)	~586 000	32.8
*Dasytastes*	sp. 01	USA, CA	Yes	Yes	Yes		Yes	Yes	600 652	33.8
*Dasytastes*	sp. 02	USA, CA	Yes	Yes	Yes		Yes	Yes	592 705	33.1
*Enallonyx*	*sculptilis*	USA, CA	No	No	No		No			
*Eschatocrepis*	*constrictus*	USA, CA	No	No	No	No	No			
*Gracilivectura*	*pygidialis*	USA, CA	No	No	No	No	No			
*Listrimorpha*	*pallipes*	USA, CA	No	No	No		No			
*Vectura*	*longiceps*	USA, CA	No	No	No		No			
*Vecturoides*	sp.	USA, CA	No				No			
Vecturoides sp.										
*Danacea*	*nigritarsis* GER	Germany	Yes	Yes	Yes	Yes		Yes	497 568	28.8
*Danacea*	*nigritarsis* ITA	Italy	Yes	Yes	Yes	Yes	Yes	Yes	497 621	28.8
*Danacea*	*pallipes*	Germany	Yes	Yes	Yes					
Danacea pallipes										
*Asydates*	*grandiceps*	USA, CA	No	No	No		No			
*Asydates*	*ruficauda*	USA, CA	No	No	No		No			
*Byturosomus*	*fuscus*	USA, CA	No	No	No		No			
*Cradytes*	*serricollis*	USA, NM	No	No	No		No			
*Cradytes*	*serrulatus*	USA, AZ	No	No	No		No			
*Eudasytes*	*grandicollis*	USA, NV	No	No	No		No			
*Listropsis*	sp.	USA, CA	No	No	No		No			
*Listrus*	sp. 00	USA, CA	Yes	Yes	Yes		Yes	Yes (draft)	~511 000	32.5
*Listrus*	sp. 01	USA, AZ	Yes	Yes		Yes		Yes	440 755	28.3
*Listrus*	sp. 02	USA, CA	Yes	Yes				Yes	513 178	33.3
*Listrus*	sp. 03	USA, NV	Yes	Yes	Yes					
*Listrus*	sp. 04	USA, UT	Yes	Yes				Yes (draft)	~515 000	32.9
*Listrus*	sp. 05	USA, CA	Yes	Yes	Yes					
*Listrus*	sp. 06	USA, CA	Yes	Yes				Yes	509 826	32.6
*Listrus*	sp. 07	USA, CA	Yes	Yes				Yes (draft)	~461 000	33.3
*Listrus*	sp. 08	USA, CA	Yes	Yes	Yes					
*Listrus*	sp. 09	USA, OR	Yes	Yes				Yes	518 020	32.9
*Listrus*	sp. 10	USA, OR	Yes	Yes	Yes	Yes				
*Microasydates*	*santabarbara*	USA, CA	No	No	No		No			
*Microasydates*	*umbratus*	USA, CA	No	No	No		No			
*Pseudasydates*	*explanatus*	USA, CA	No	No	No		No			
*Pseudasydates*	sp.n.	USA, NM	No	No	No		No			
*Trichochrous*	*aenescens*	USA, CA	No	No	No		No			
*Trichochrous*	*brevicornis*	USA, CA	No	No	No		No			
*Trichochrous*	*convergens*	USA, AZ	No	No	No		No			
*Trichochrous*	sp.n.	USA, CA	No	No	No		No			
*Trichochrous*	*egenus*	USA, CA	No	No	No		No			
*Trichochrous*	*fulvotarsis*	USA, CA	No	No	No		No			
*Trichochrous*	*fulvovestitus*	USA, CA	No	No	No		No			
*Trichochrous*	*pallescens*	USA, CA	No	No	No	No	No			
*Trichochrous*	*quadrinotatus*	USA, CA	No	No	No		No			
*Trichochrous*	*seriellus*	USA, UT	No	No	No		No			
*Trichochrous*	*sordidus*	USA, CA	No	No	No		No			
*Trichochrous*	sp.n. Trich076	USA, CA	No	No	No		No			
Trichochrous sp.n.Trich076										
*Dasytes*	*aeratus*	Germany	Yes	Yes	Yes		Yes	Yes	455 894	30.9
*Dasytes*	*alpigradus*	Italy	Yes	Yes	Yes			Yes	476 890	30.0
*Dasytes*	*niger*	Germany	Yes	Yes	Yes	Yes	Yes	Yes	486 045	30.9
*Dasytes*	*plumbeus*	Germany	Yes	Yes	Yes	Yes	Yes	Yes	456 731	30.4
*Dasytes*	*subaeneus*	Germany	Yes	Yes	Yes					
*Dasytes*	*tristiculus*	Italy	yes	yes	yes	yes	yes			
*Dasytes*	*virens*	Germany	yes	yes	yes	yes	yes	yes	456 754	30.4
*Dolichosoma*	*lineare*	Germany	yes	yes	yes	yes				
*Psilothrix*	*viridicoerulea*	Germany	yes	yes	yes	yes	yes	yes	464 075	28.7

### DNA extraction

Head and leg tissue samples taken from the insects were extracted for the identification of host species. For diagnostic PCRs for the presence of *Dasytiphilus*, bacterial community profiling, and genome sequencing, either dissected bacteriomes or, if not possible, entire host abdomens were extracted in order to obtain bacterial symbiont DNA (Fig. S1). Extractions were done using the Epicentre MasterPure™ Complete DNA and RNA Purification Kit (Illumina Inc., Madison, WI, USA) following the manufacturer’s instructions, including RNase digestion. DNA of samples intended for genome sequencing had its purity assessed using a NanoPhotometer P330 (Implen, München, Germany) and DNA quantity was measured using a Qubit dsDNA BR assay kit on a Qubit 2.0 Fluorometer (Invitrogen by Thermo Fisher Scientific, MA, USA).

### Diagnostic PCR and Sanger sequencing to assess the presence of *Dasytiphilus*

General eubacterial and diagnostic PCRs were performed to assess *Dasytiphilus* presence and were done on a T-Professional-Gradient Thermocycler (Biometra, Göttingen, Germany) using reaction mixes of 6.9 μl ultrapure H_2_O, 1.25 μl of 10× reaction buffer, 0.25 μl 25 mM MgCl_2_, 1.5 μl 2 mM dNTPs, 1 μl of both forward and reverse primer (each 10 pmol/μl), 0.1 μl of 5 U/μl Taq DNA polymerase and 1 μl template. Alternatively, a Mastercycler EP Gradient S Thermocycler (Eppendorf AG, Hamburg, Germany) was used with a reaction mix containing 9.5 μl ultrapure H_2_O, 12.5 μl of Q5® High-Fidelity 2X Master Mix (NEB), 1 μl of both forward and reverse primer (each 10 pmol/μl), and 1 μl template. Primers used for *Dasytiphilus* identification were the eubacterial primer pairs fD1 and rP2 as well as slightly modified versions fD1_Mely and rP2_Mely. Moreover, the symbiont-specific primer pair Dasy_Sym_fwd3 and Dasy_Sym_rev3 was utilized (Table S1).

To guarantee that *Dasytiphilus* sequences were amplified, Sanger sequencing was conducted following general eubacterial and diagnostic PCRs. Products were purified using the innuPREP PCRpure Kit (Analytik Jena, Jena, Germany) according to the manufacturer’s instructions and were subsequently sequenced by a commercial service (StarSeq, Mainz, Germany). Alternatively, products were purified with the Zymo Research DNA Clean & Concentrator-5 kit (Zymo Research, Irvine, CA, USA) following the manufacturer’s protocol and were sequenced in house with an AB Hitachi 3730XL DNA Analyzer (Applied Biosystems by Thermo Fisher Scientific, MA, USA).

### Microbial community profiling by 16S rRNA gene amplicon sequencing

Bacterial community composition was evaluated via high-throughput amplicon sequencing of the 16S rRNA gene. Samples were sequenced in a paired end approach with read lengths of 300 nt by a commercial provider (StarSeq, Mainz, Germany) on an Illumina MiSeq platform (Illumina Inc., San Diego, CA, USA) using V3 reagent and 25% PhiX to balance the composition of bases. Amplified regions were either V3–V4 region with primers 341f and 806bR, V4 region with primers 515F and 806bR or V4–V5 region with primers 515F and 909R (Table S1). This was due to temporal differences in sample availability and constraints in assay design due to concurrent sequencing projects. A comprehensive list of all sequenced Melyridae samples with the amplified region can be found in Table S2. No-template DNA extractions were sequenced as negative controls. Based on the sequenced Illumina reads, amplicon sequence variants (ASVs) were obtained after read trimming depending on the amplified region, quality filtering, dereplicating, and chimera removal in R utilizing the package DADA2 [38]. Subsequently, the pretrained classifier Silva 138.1 was used to assign taxonomy [39, 40]. Finally, reads identified as chloroplast or mitochondria were removed prior to analysis.

### Symbiont localization via fluorescence *in situ* hybridization

To localize the symbionts in multiple species of Melyridae beetles, FISH was performed on specimens of 13 different Dasytinae species and *M. bipustulatus* as an outgroup (Table 1). Whole beetles or abdomens were individually fixed in Carnoy’s solution (67% ethanol, 25% chloroform, and 8% glacial acetic acid), dehydrated in a series of ascending concentration (30%, 50%, 70%, 80%, 90%, and 96%) of *n*-butanol and subsequently embedded in Technovit 8100 (Heraeus Kulzer) following the manufacturer’s instructions. Transversal or sagittal histological sections of 8 μm thickness were prepared utilizing a glass knife on a Leica RM 2245 microtome and transferred to microscope slides. To each slide, 100–150 μl hybridization mix was applied and then covered with a glass cover slip. The hybridization mix contained hybridization buffer (0.9 M NaCl, 0.02 M Tris/HCl (pH = 8), 0.01% SDS), 0.5 μM of the fluorescently labeled oligonucleotide probes EUB338-Cy3 and Dasy_ent_Cy5 (Table S1), and 0.5-mg/ml DAPI for host cell counterstaining. Slides were hybridized overnight at 50°C in a humid chamber. Afterwards, the glass cover slip was removed and the slides were washed at 50°C for 2 h submerged in wash buffer, followed by an additional washing step in distilled water at 50°C for 20 min. The wash buffer consisted of 0.1-M NaCl, 0.02-M Tris/HCl (pH = 8), 5 mM EDTA, and 0.01% SDS. After washing, 30 μl of VectaShield was applied to each slide upon which a glass cover slip was then placed. Samples were observed with a Thunder Imaging System (Leica, Wetzlar, Germany). The signals for Cy3, Cy5, background, and DAPI were acquired with the 555-, 635-, 475-, and 390-nm EFW LED8 respectively, at 50% (Cy3, Cy5, background) power or 5% power (DAPI), the DFT51010 filter cube, and a 590-, 642-, 535-, and 460-nm fast emission filter, respectively. Finally, images were processed in the Leica Application Suite X software (Leica, Wetzlar, Germany) with the instant and small volume computational clearing algorithms.

### Symbiont genome sequencing and assembly

For symbiont genome reconstruction, short-read shotgun library preparation and sequencing was performed by the Max Planck Genome Center (Cologne, Germany) utilizing a HiSeq 3000 or NextSeq 2000 system (Illumina Inc., San Diego, CA, USA) or by a commercial service (CeGaT GmbH, Tübingen, Germany) using a NovaSeq 6000 platform (Illumina Inc., San Diego, CA, USA) (Table S3). Obtained paired sequence reads of 150 bp each were uploaded to KBase [41], and read quality was assessed with FastQC v0.11.5-v0.11.9. The reads were trimmed using Trimmomatic v0.36 [42], and trimmed reads were assembled with metaSPAdes v3.13.0-v3.15.3 [43] and MEGAHIT v1.2.9 [44].

For *Listrus* sp. 02, long reads were used in addition to the Illumina reads to assemble the symbiont genome. For this, high-molecular-weight (HMW) genomic DNA was selectively extracted with the Short Read Eliminator Kit XS (Circulomics, MD, USA), to enrich for fragments longer than 10 kb. The size-selected HMW DNA was used as the starting material for the preparation of an ONT library, following the manufacturer’s guidelines for the Ligation Sequencing Kit SQK-LSK110 (Oxford Nanopore Technologies, Oxford, UK), and sequenced on R9.4.1 (FLO-MIN106) flow cells using the MinION sequencing device with MinKNOW Software version 22.10.7. Super high-accuracy (SUP) base-calling was performed using GUPPY version 5.0.14. Flye (v2.9.1) assembler [45, 46] in meta-mode was used to generate a *de novo* genome assembly from the ONT data, followed by four iterations of polishing using Racon and one round of error correction using Medaka. For final error correction of the ONT-based assembly, we used ntEdit with paired-end (2× 150 bp) Illumina data generated from the same HMW DNA used for ONT sequencing. To remove duplications (heterozygous regions) and generate haploid genome(s) for further downstream analysis, we used purge_haplotigs.

### Symbiont genome analysis

After assembly, the received contigs were annotated with Prokka v1.14.5 [47] and Rast v1.073 [48]. Symbiont contigs were identified in Geneious (Geneious Prime various versions between 2019 and 2023) based on length, GC content, read coverage, and gene synteny. Contigs from both assembly methods were *de novo*-assembled, resulting in either closed genomes or high-quality draft genomes. Minor polishing was done on selected genomes by read mapping and pilon genome improvement [49]. Genomes were analyzed with the help of KEGG: Kyoto Encyclopedia of Genes and Genomes [50–52]. Gene synteny was visualized with Clinker [53], showing only the highest similarity links between genes. When analyzing specific symbiont capabilities, we focused on pathways that could be directly relevant to the interaction with the host.

### Symbiont phylogenetic reconstruction

The symbiont phylogeny was reconstructed in KBase [41] with the plugin “Insert Set of Genomes Into SpeciesTree – v2.2.0.” The plugin took user-provided genomes and constructed alignments of 49 core, universal genes defined by COG (Clusters of Orthologous Groups) gene families with publicly available genomes of closely related bacteria. Subsequently, it used an approximately-maximum-likelihood algorithm to create a phylogenetic tree [54].

### dN/dS of lysine pathway genes

We evaluated the selective forces acting on lysine pathway genes of *Listrus* spp. more closely by examining the ratio of synonymous vs nonsynonymous substitutions in order to assess whether substitution patterns differed from those of lysine pathway genes of *Dasytastes* clade symbionts. To infer orthologous genes, orthogroups were reconstructed using OrthoFinder v2.5.4 [55] with the MSA option enabled using muscle v5.1 [56] and FastTree v2.11.1 [54]. Orthologs for each of the candidate genes were extracted from the phylogenetic hierarchical orthogroups results and used for downstream analysis. For each candidate gene, orthologous amino acid sequences were aligned with muscle v5.1 with default parameters. Then, the corresponding nucleotide sequences were codon-aligned based on the protein alignments using the pal2nal v14 script [57]. Phylogenetic trees were reconstructed using FastTree v2.11.1 based on codon alignments. Both tree and alignment were used to detect changes in the selective pressures acting on *Listrus* spp. genes compared to the outgroup. Branches corresponding to *Listrus* spp. were marked using the vision.hyphy.org tool and the “select descendant branches” enable. Eventual relaxation or intensification of the selection strength was tested using RELAX from HYPHY package v2.5.33 [58]. Additionally, a test for positive selection was conducted using aBSREL [59] from HYPHY package. The *P-*value threshold for considering tests as significant was set at 0.05.

### Host identification and phylogenetic reconstruction

#### Host species identification

To robustly link host species with symbiont presence/absence, we attempted to identify each analyzed host taxon. Host species that could not be identified based on morphology were identified via DNA barcoding, i.e. sequencing of the cytochrome c oxidase subunit I gene (CO1). In this gene, >2% sequence divergence is usually recorded between closely related animal species, so the mutation rate is high enough to distinguish between species [60]. The CO1 gene was amplified using PCR with primers LepF1 and LepR1 (Table S1), and the product was purified and subjected to Sanger sequencing (see above for details). However, several species could not be identified to a described species because no conspecific reference sequence was available and the necessary modern taxonomic revisions have not been completed. For the genus *Listrus*, in particular, species are poorly characterized and undescribed species certainly exist; as a consequence, only morphospecies designations were given for members of this genus.

#### Host phylogenetic reconstruction

To infer the evolutionary history of the symbiosis with *Dasytiphilus,* we reconstructed a phylogeny of Dasytinae beetles and outgroup taxa based on the sequences of up to four marker genes: 18S rRNA gene, 28S rRNA gene, CO1, and Cytochrome b gene (cytB). Where possible, sequences were obtained from the assembled shotgun sequencing data; otherwise, the respective regions were amplified by PCR and Sanger-sequenced. The partial 18S rRNA gene (~1400 bp) was amplified with primer pair 18S_ai and 18S_3’I. Three individual pieces were sequenced with primers 18S_ai, 18S_a1.0, and 18S_3’I, respectively, and subsequently assembled to cover the complete amplified region. The partial 28S rRNA gene (~600 bp) was amplified with primer pair 28Sff and 28Srr and sequenced with 28Sff. For the CO1 gene, either one long sequence (~1400 bp) was amplified with primer pair LepF1 and Pat or two smaller overlapping fragments were amplified, the first one with primer pair LepF1 and LepR1 and the second one with primer pair Melyridae_CO1_F1 and Pat. Following this, three pieces were sequenced with primers LepR1, Melyridae_CO1_F1 and Pat and assembled to obtain a contiguous sequence. The partial cytB gene (~300 bp) was amplified with primer pair Sytb_F and Sytb_R and sequenced with Sytb_F. Additionally, previously published sequences for some species were used [28]. For each gene, an alignment was created using Geneious Prime 2023.1.2. The four alignments were concatenated and used for phylogenetic reconstruction via Bayesian analysis [61] utilizing the MrBayes plugin v3.2.6 in Geneious Prime 2023.1.2. Through the custom command block, the alignment was partitioned by gene, and for the protein-coding genes CO1 and cytB, also by codon position (codon positions 1 and 2 in one partition, position 3 in another partition). From a random starting tree, two runs with four chains were run over 20 million generations, until the standard deviation of split frequencies was consistently below 0.01, using a 4by4 GTR model and gamma-shaped rate variation. Sampling was done every 2000 generations, and a consensus tree was created after 25% burn-in.

## Results

### *Dasytiphilus* is widespread but not omnipresent in Dasytinae beetles

We screened 58 Melyridae species (Table 1), mostly from the subfamily Dasytinae, for the specific symbiont *Dasytiphilus*, confirming the presence of *Dasytiphilus* in 27 Dasytinae species via multiple different methods, whereas no symbionts were detected in 29 other Dasytinae species nor in the two investigated species of the subfamily Malachiinae. Additionally, *Dasytiphilus* was also not detected in *Eronyxa pallida* from the closely related beetle family Lophocateridae.

**Figure 1:**
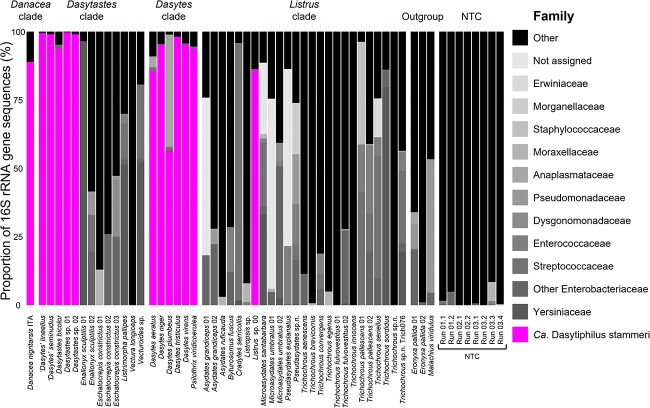
Bacterial community composition in Melyridae beetles given in relative abundance of bacterial ASVs determined at family level by DADA2 analysis of Illumina 16S rRNA gene amplicons. Samples with <1000 reads after removal of reads assigned to chloroplasts and mitochondria were excluded. Every bar represents a single individual, with DNA extracted from the whole body. The Dasytinae-specific symbiont *Dasytiphilus* is highlighted in magenta. Only the 50 most abundant bacterial ASVs are displayed with annotated family, remaining ASVs are grouped as “Other.” NTC, no template extraction controls.

The 16S rRNA gene amplicon sequencing analysis of a subset of the taxa revealed that *Dasytiphilus* was present in 13 and absent in 30 Melyridae species (Fig. 1, Fig. S2). When symbionts were present, they always constituted over 50% of the total microbial community (range: 56%–99%), thus making false-negative results unlikely.

### *Dasytiphilus* is present in distinct host clades and codiversified with its hosts

**Figure 2:**
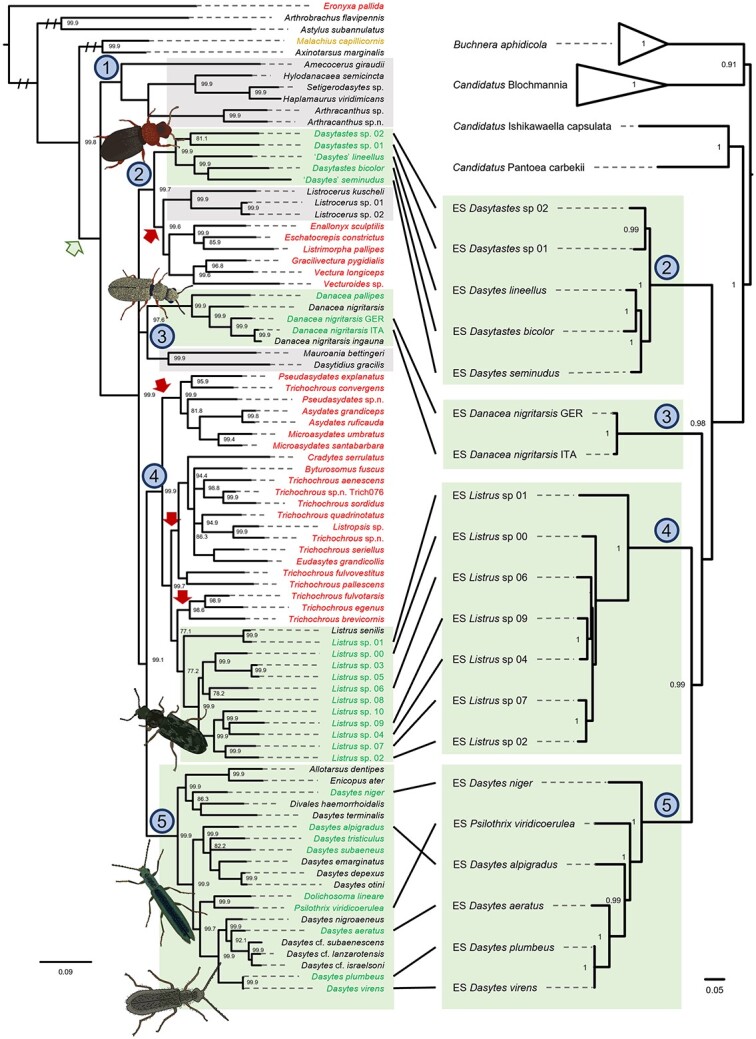
Evolutionary history of Dasytinae beetles and their endosymbiont (ES) “*Candidatus* Dasytiphilus stammeri”. The phylogeny on the left shows the relationships of Melyridae beetles based on a partitioned Bayesian analysis of COI, cytb, 18S, and 28S genes. Five distinct clades were evident in the Dasytinae: (1) the “*Amecocerus*-clade;” (2) the “*Dasytastes*-clade;” (3) the “*Danacea*-clade;” (4) the “*Listrus*-clade;” and (5) the “*Dasytes*-clade.” Taxa in green font carry the Dasytinae-specific symbiont *Dasytiphilus*, whereas no evidence for the symbiont’s presence was found for taxa in red font. Taxa in black font were not screened for the symbiont, but were added to the phylogeny to better resolve phylogenetic relationships. Although *M. capillicornis* in particular was not screened for the symbiont, the orange font indicates that other *Malachius* species were investigated and showed no signs of *Dasytiphilus* presence. Green and red arrows indicate potential symbiont acquisition and loss events, respectively, during the evolutionary history of the Melyridae. Green boxes highlight clades in which all screened species harbored *Dasytiphilus* and in which presumably all host taxa are colonized. Gray boxes indicate clades for which the symbiont status of the host species is unknown. Node labels indicate support values, based on Bayesian posterior probabilities, with values below 75 removed. Beetle illustrations depict representative species of the symbiont-containing clades, i.e. *Dasytastes bicolor*, *Danacea nigritarsis*, *Listrus* sp., *Dolichosoma lineare*, and *D. plumbeus* (from top to bottom). The right side depicts the phylogenetic relationships of the different *Dasytiphilus* strains based on a set of 49 COGs. The phylogeny was reconstructed using an approximately-maximum-likelihood algorithm, and node labels indicate local support values. Outgroups are the close relatives “*Candidatus* Pantoea carbekii”, “*Candidatus* Ishikawaella capsulata”, *B. aphidicola*, and “*Candidatus* Blochmannia” endosymbionts. Connections between the phylogenies highlight host–symbiont associations.

On the basis of four marker genes, we reconstructed a phylogeny of Dasytinae beetles and representative taxa from the subfamilies Melyrinae and Malachiinae (Fig. 2). In our analysis, the Dasytinae formed a monophyletic clade, slightly differing from a previously published topology [28], in which the Melyrinae were nested within Dasytinae. Five distinct clades were evident in Dasytinae: (i) the “*Amecocerus*-clade,” represented by five Southern Hemisphere genera; (ii) the “*Dasytastes-*clade,” represented by seven North American genera (and including species currently misplaced in *Dasytes*) and one South American genus (*Listrocerus*); (iii) the “*Danacea*-clade,” represented by three Palearctic genera; (iv) the “*Listrus*-clade,” represented by nine nominal North American genera; and (v) the “*Dasytes*-clade,” represented by six Palearctic genera. These five clades correspond to putative natural tribes within Dasytinae based on morphology and contradict current nominal tribal assignments in several cases (e.g. four of the *Amecocerus*-clade genera are currently placed with *Danacea* in the Danaceini). Historical generic and tribal placement based on superficial morphological traits often does not reflect the actual relationships within the Dasytinae. The subfamily is currently undergoing revision and reclassification (e.g. see [62]). Hence, several genera represented here will be either transferred to new or different tribes (e.g. all *Amecocerus*-clade and *Dasytastes*-clade genera) or synonymized with other genera (e.g. *Byturosomus*, *Cradytes*, *Eudasytes*, *Listropsis*). Furthermore, certain species in the phylogeny will either be transferred (e.g. the “*Dasytes*” species in the *Dasytastes*-clade) or are as-yet-undescribed species (e.g. *Pseudasydates* sp.n., *Trichochrous* sp.n.). Beetles belonging to the *Dasytastes*-clade and the *Listrus*-clade were collected in North America, whereas beetles in the *Danacea*-clade and the *Dasytes*-clade originated from Europe. Species harboring *Dasytiphilus* clustered in the last four of the aforementioned clades, although no *Amecocerus*-clade species were screened. All analyzed European Dasytinae species harbored *Dasytiphilus*. In contrast, we could not detect the symbiont in several of the North American taxa.

**Figure 3:**
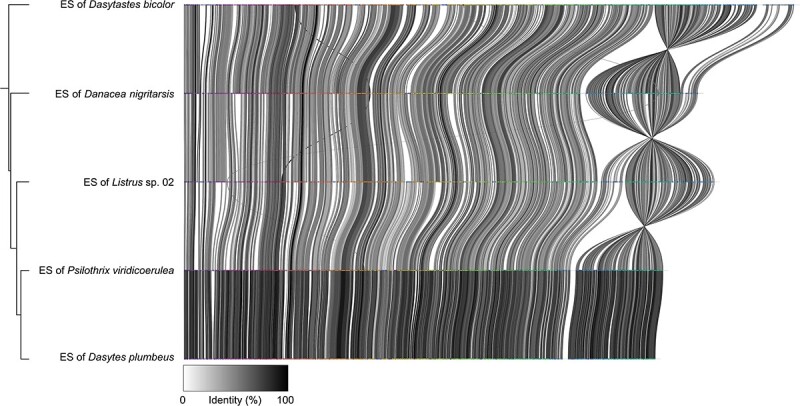
Genome synteny of *Dasytiphilus* representatives from each colonized host clade. Gene synteny plot, comparing the gene order between the *Dasytiphilus* endosymbionts of *Dasytastes bicolor* (*Dasytastes*-clade), *D. nigritarsis* ITA (*Danacea*-clade), *Listrus* sp. 02 (*Listrus*-clade), *Psilothrix viridicoerulea* (*Dasytes*-clade), and *D. plumbeus* (*Dasytes*-clade). The gene identity percentage of homologous proteins is based on amino acid sequences and indicated by different gray values. The phylogeny on the left is based on a set of 49 COG and was reconstructed using an approximately-maximum-likelihood algorithm.

The symbiont phylogeny was created based on 49 marker genes extracted from whole genomes or almost complete draft genomes (Fig. 2, Fig. S3). The symbionts’ closest relatives were “*Candidatus* Ishikawaella capsulata,” *Buchnera aphidicola*, and “*Candidatus* Blochmannia” endosymbionts. The different *Dasytiphilus* strains together formed a well-supported monophyletic clade, and the symbionts’ phylogenetic relationships broadly corresponded to those of their hosts, with minor discrepancies within the four major clades. The 16S rRNA gene nucleotide similarity between different symbionts within each clade was high (within *Dasytastes-*clade 97.8%–99.3%; within *Danacea-*clade 96.0%–99.7%; within *Listrus-*clade 97.3%–99.8%; within *Dasytes-*clade 95.2%–99.9%), whereas the similarity between clades was in the range of 90%–94%, regardless which clades were compared. Based on this 16S rRNA gene similarity, the symbionts from different clades could be considered distinct species, with multiple strains within each clade. However, given their similar ecological niche within Dasytinae beetle hosts and to avoid confusion, we recommend using the previously published name “*Candidatus* Dasytiphilus stammeri” [36] for all *Dasytiphilus* symbionts of Dasytinae beetles. Host species affiliation can then be indicated by strain names using a four-letter code, consisting of the first letter of the host genus name and the first three letters of the host species epithet (e.g. “*Candidatus* Dasytiphilus stammeri” DPLU for the *Dasytiphilus* symbiont of *D. plumbeus*) (see [13]). The similarity of symbiont sequences was also reflected in the high gene synteny between the different *Dasytiphilus* strains, with symbionts within each clade exhibiting perfect gene synteny (Fig. S4), whereas individual rearrangements were observed between clades (Fig. 3).

### *Dasytiphilus* is located intracellularly in bacteriomes

**Figure 4:**
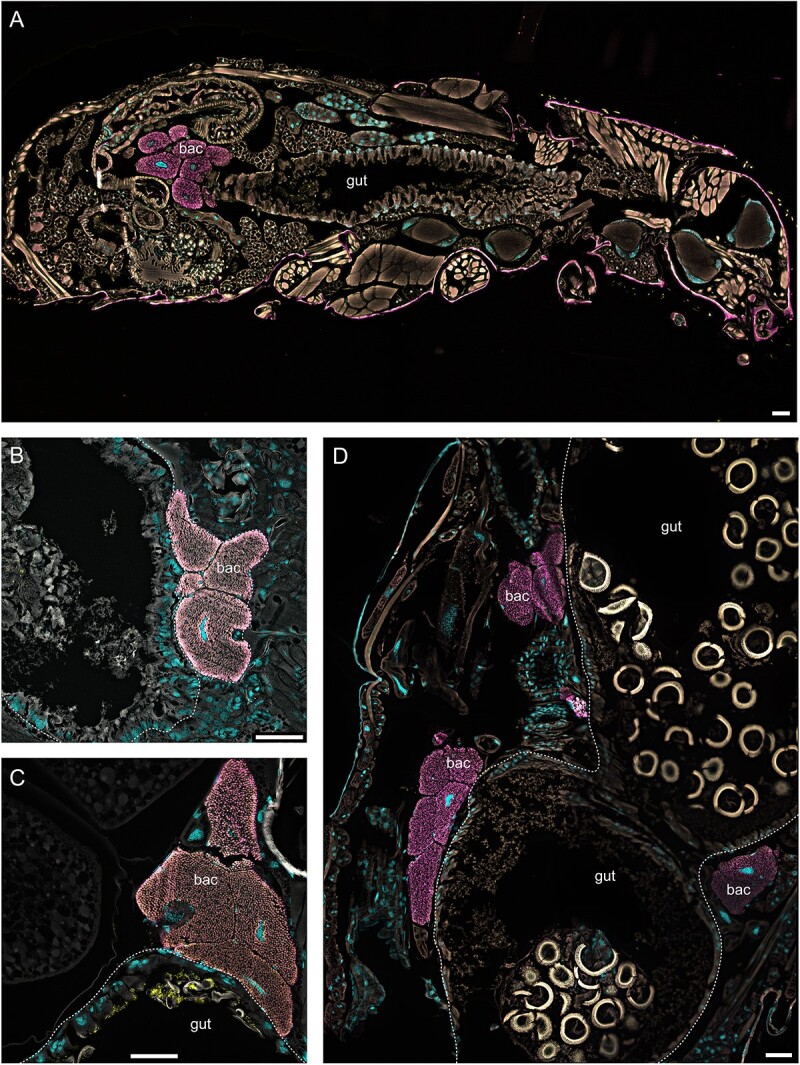
Tissue localization of *Dasytiphilus* symbionts in the abdomen of *Listrus* sp. 01 (A), *D. nigritarsis* GER (B), *D. plumbeus* (C), and *D. tristiculus* (D), as revealed by FISH sagittal sections. *Dasytiphilus* (in magenta) are densely packed in the bacteriocytes (bac) that were located in close proximity to the gut and the Malpighian tubules at the midgut-hindgut junction in adults of all analyzed species. General bacteria (in yellow) are sometimes visible in the gut (C). Background autofluorescence is given in white and a general DNA counterstain in cyan (DAPI). Pictures shown are overlaps of all four channels. Consumed pollen was often visible in the gut (C and D, with a strong auto-fluorescence in the Cy3 channel), confirming the pollen-feeding habit of the beetles. Scale bars = 50 μm.

By performing FISH using symbiont-specific and general eubacterial probes on histological sections of adult beetles, *Dasytiphilus* was localized intracellularly in bacteriomes in various Dasytinae (Fig. 4, Fig. S5). The bacteriomes are located in close proximity to the gut and the Malpighian tubules at the midgut-hindgut junction in adults of all analyzed species. Outside of the bacteriomes, no *Dasytiphilus* were found, including the ovaries (Fig. S6), with the exception of a single event in which the symbionts were localized in the gut (Fig. S7). Furthermore, *Dasytiphilus* could not be detected in any of the analyzed non-Dasytinae Melyridae, nor in several Dasytinae belonging to clades in which the symbionts were also not detected by PCR and sequencing (Fig. S8).

### Symbiont functional capabilities and genome evolution

**Figure 5:**
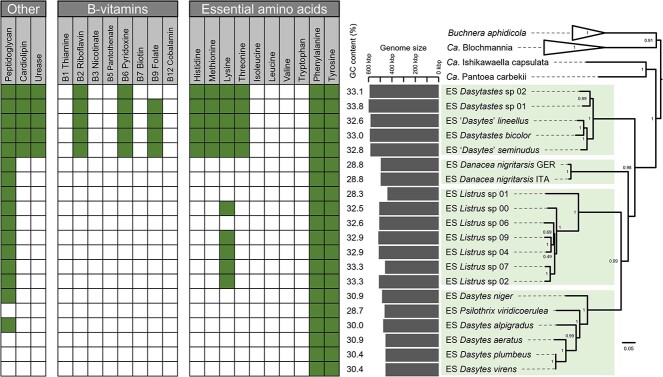
Overview of selected metabolic capabilities of *Dasytiphilus* strains. Presence/absence of selected metabolic pathways (cell envelope and nitrogen recycling, B-vitamin biosynthesis, essential amino acid biosynthesis) based on genomic data for *Dasytiphilus* strains of 20 host species. Green squares indicate the presence of a putative functional pathway, empty squares show the absence of the pathway. Symbiont phylogeny on the right is the same as in Fig. 2, depicting the phylogenetic relationships between the different symbiont strains. Genome length (gray horizontal bars) and GC content are given for all assembled *Dasytiphilus* strains.

To gain insights into the functional capabilities of *Dasytiphilus* and its evolution, the genomes of symbionts associated with 20 different host species were sequenced, assembled, and analyzed. Symbiont genome sizes varied between 437 and 601 kb, and GC content ranged from 28.3% to 33.8% (Fig. S9), and both correlated strongly (Spearman rank correlation: *S* = 220.66, rho = 0.83, *P* value <0.0001) (Fig. S10). Coding density ranged from 84% to 93% and fell into the range of other endosymbionts [22]. Differences in symbiont genome lengths were also reflected in their metabolic capabilities (Fig. 5, Fig. S11); however, some capabilities were universally retained. All *Dasytiphilus* strains retained glycolysis and pentose phosphate pathways, both utilizing ß-D-Fructose-6P as the starting metabolite. However, the citrate cycle was incomplete, as the symbionts only encode genes for the enzymatic steps from 2-oxoglutarate to oxaloacetate via succinate. Similarly, the genetic repertoire for housekeeping, i.e. DNA replication and repair, transcription, and translation, was also reduced (Table S4).

Even though the *Dasytiphilus* strains featured heavily eroded genomes, several potentially host-relevant capabilities were retained (Supplementary File 02). All *Dasytiphilus* strains encoded genes for the complete shikimate pathway and subsequent tyrosine biosynthetic pathway (*aroF*/*G*/*H*, *aroB*, *aroQ*, *aroE*, *aroK*/*L*, *aroA*, *aroC*, *tyrA*, and *aspC*) (Fig. S11). Furthermore, all *Dasytastes*-clade symbionts and several *Listrus*-clade symbionts had almost complete lysine biosynthesis diaminopimelate pathways. Even though none of the strains had a fully complete lysine pathway (Fig. S11), we postulate that it is indeed functional in all *Dasytastes*-clade symbionts and several *Listrus*-clade symbionts, hypothesizing that the missing catalytic steps encoded by *argD*/*dapC* (missing in *Dasytastes*-clade and *Listrus*-clade symbionts), *lysA*, and *dapE* (both absent in *Listrus*-clade symbionts) are likely taken over by multifunctional enzymes or encoded by the host. For example, a phosphoserine aminotransferase encoded by the *serC* gene can compensate for the catalytic step usually performed by the enzyme encoded by the *argD/dapC* gene [63]*.* Concordantly, the *serC* gene was present in all analyzed *Dasytastes* and *Listrus* symbionts that otherwise had a nearly complete lysine synthesis pathway, whereas it was missing in *Danacea* and *Dasytes* symbionts that lacked the lysine pathway. The absence of *lysA* and *dapE* is likely compensated for by other symbiont or host enzymes, as hypothesized for other insect endosymbionts [64–66] (see Supplementary Results for more detailed information). Symbionts from the *Dasytastes*-clade also encoded genes for the pathways of the essential amino acids histidine, methionine, and threonine.

Beyond amino acids, *Dasytastes*-clade symbionts encoded genes for incomplete but likely functional biosynthetic pathways of the B vitamins riboflavin (B2), pyridoxine (B6), and folate (B9). A missing step in the enzymatic pathway for riboflavin synthesis can be taken over by an enzyme encoded by *yigL* [67], which was found in all *Dasytastes*-clade symbionts but missing in all other *Dasytiphilus*. Additionally, symbionts from the *Dasytastes*-clade are likely able to synthesize vitamin B6, and the absence of *dxs* could be compensated for by the presence of *dxr*, which encodes DXP reductoisomerase [68]. Moreover, symbionts of the *Dasytastes*-clade, with the exception of *Dasytastes* sp. 02, are presumably able to synthesize the vitamin B9 folate (see Supplementary Results for more detailed information).

Besides biosynthetic pathways encoding metabolites that can be beneficial for the host, further differences between the symbiont strains existed. Symbionts from the *Dasytastes*-clade encoded genes for urease (encoded by *ureA*, *ureB*, and *ureC*) and its auxiliary proteins (encoded by *ureD*/*ureH*, *ureF*, and *ureG*), which catalyzes the hydrolysis of urea [69]. A missing auxiliary protein encoded by *ureE* is not essential for the functioning of the urease [70, 71]. The urease-catalyzed hydrolysis of urea by *Dasytastes*-clade symbionts likely provides ammonia that the bacterium could use to synthesize glutamine with the help of glutamine synthetase encoded by *glnA* [72, 73]. Subsequently, glutamine could be further processed to carbamoyl phosphate by an enzyme complex encoded by *carA* and *carB* [74]. Furthermore, the symbiont strains differed in their capabilities to synthesize metabolites important for the cell envelope. Symbiont strains of the *Dasytastes*-clade were the only strains able to produce cardiolipin. Additionally, symbionts of *P*. *viridicoerulea*, *D. aeratus*, *D. plumbeus*, and *D. virens* lost the pathway to synthesize peptidoglycan.

#### Evolutionary trajectories of lysine pathway genes

The presence or absence of most biosynthetic pathways and other capabilities were homogeneous within the different clades. However, there were sporadic exceptions, e.g. the symbiont of *Listrus* sp. 01 lost 12 NADH-quinone oxidoreductase subunit genes. The lysine biosynthesis pathway was in varying states in the *Listrus*-clade symbionts: partial but functional in most *Listrus*-clade symbionts, nonfunctional because of pseudogenized genes in *Listrus* sp. 06, or completely absent in *Listrus* sp. 01. In order to gain insights into the evolutionary trajectories of these genes in *Listrus*-clade symbionts, we performed a dN/dS analysis on genes involved in lysine biosynthesis. Several genes (*dapA*, *dapE*, *dapF*, and *thrA*) displayed significant relaxation of purifying selection in *Listrus* symbionts compared to *Dasytastes*-clade symbionts (Supplementary File 03); however, those genes were still under purifying selection.

## Discussion

Mutualistic insect–bacterial symbioses are common, with symbionts providing a large array of benefits to their hosts. Here, we report on a widespread bacterial endosymbiont within the Dasytinae subfamily of the Melyridae and elucidate its evolutionary history and dynamics. Host and symbiont phylogenies indicate not only long-term coevolution that originated from a single symbiont acquisition but also several independent loss events. A comparison of 20 symbiont genomes revealed that extensive genome erosion occurred early in the evolutionary history of the symbiosis but also identified core genes retained by all symbiont strains. The symbionts’ main contribution to their hosts is likely the synthesis and provision of tyrosine to support cuticle formation, which is, to our knowledge, the first reported case of tyrosine supplementation in insects with pollen-feeding adults. Other potential supplementation in the form of essential amino acids, B vitamins, and nitrogen recycling likely play a role in some but not all of the taxa.

### Evolution of the symbiosis with *Dasytiphilus*

Comparing host and symbiont phylogenies provides strong evidence for codiversification between the two symbiotic partners (Fig. 2). Furthermore, it is likely that *Dasytiphilus* was acquired from a single event, considering the monophyly of the symbiont clade and the high degree of gene synteny between the different symbiont strains (Fig. 3, Fig. S4). The symbiont was not found outside of the Melyridae nor in the examined Malachiinae beetles *M. viridulus* and *M. bipustulatus*. Furthermore, Buchner [9] did not report that any Melyridae outside of the Dasytinae have a bacteriome, even though it is very likely that he had access to common species from other subfamilies. Thus, the symbiosis with *Dasytiphilus* likely originated in the ancestor of the Dasytinae or slightly later after the separation of the *Amecocerus*-clade, the earliest-branching clade of Dasytinae. Critical to determining the origin will be the screening of species from the *Amecocerus*-clade, as well as members of the subfamily Melyrinae, which were recovered as the sister-group to the *Amecocerus*-clade in previously published work [28] but sister to Malachiinae + Dasytinae in this study, and the other families of the melyrid lineage (Prionoceridae, Rhadalidae, Mauroniscidae, Phycosecidae). Since the origin of the symbiosis, multiple North American lineages lost *Dasytiphilus* (Fig. 2). It is conceivable that symbiont acquisition as well as symbiont losses coincided with lifestyle changes. Adult beetles from the subfamily Dasytinae are known to feed on pollen [29], whereas the larvae are reported to be scavengers or predators [26, 31, 32]. Conversely, members of other Melyridae subfamilies feed on arthropods throughout their entire life, sometimes being active hunters [37]. Although some pollen offer a wide range of amino acids, some lack certain amino acids like tyrosine [34, 35], and plant material is generally lower in nitrogenous compounds than animal tissues [75]. The acquisition of *Dasytiphilus* may have facilitated a shift from predatory or scavenging lifestyle to a diet enriched in plant material by compensating nutritional deficiencies via the supplementation of amino acids and vitamins as well as the capacity to recycle urea. Unfortunately, detailed information on the natural history of North American Dasytinae is lacking, so the ecological factors explaining the loss of the symbionts and compensating for the ensuing nutritional deficiencies remain unknown. We have found no evidence of symbiont replacement or acquisitions of co-obligate symbionts, but individual events cannot be completely ruled out. Furthermore, the gut microbiota can supplement the nutritional needs of hosts, and may make a specialized symbiont obsolete. Future research is needed to expand the understanding of potential roles of other Dasytinae-associated microorganisms.

### Tyrosine provisioning as a conserved function of *Dasytiphilus*

All analyzed *Dasytiphilus* strains retained the capability to synthesize tyrosine, so it seems likely that the provisioning of this aromatic amino acid is the core function of the symbionts. Tyrosine is a pivotal precursor for metabolites necessary for the melanization and sclerotization of the cuticle [76, 77]. Consequently, the availability of tyrosine directly impacts the physicochemical properties of the cuticle [78]. A lack of tyrosine can lead to a formation of a thinner, softer cuticle that is impaired in providing protection against biotic and abiotic stresses [11, 12, 17, 19]. Given that Dasytinae often occur in arid and semi-arid regions [26], a thick cuticle is likely important to protect the beetles against desiccation. Furthermore, their cuticle could be an effective defense or deterrent against predation, whereas symbiont-free Malachiinae evolved chemical defenses and aposematic coloration instead of relying on their poorly sclerotized and much weaker cuticle [79, 80]. The observation that in some *Dasytes* and *Psilothrix* species the bacteriomes seem to regress with age in adults [81] supports the hypothesis that the main contribution of the symbionts is completed as soon as the cuticle is formed [17]. The tyrosine demands of a beetle peak during (late) larval and pupal stages, before the adult cuticle is about to be developed. Unfortunately, knowledge of the larval diet, and thus, dietary tyrosine availability, is absent for most genera of Dasytinae. Only for some Palearctic *Dasytes* and a *Psilothrix* species has it been reported that the larvae are predators and scavengers, or change from scavengers to herbivores as they develop, respectively [31, 33, 82]. It is unlikely that any Dasytinae larvae have a pollen-based diet, based on the few circumstances of larval collection (in organic ground litter), and lack of larval collection in flowers despite countless collection events of adults in flowers (M.L. Gimmel, pers. obs.). However, it appears that the potentially tyrosine-rich diet of arthropod prey or carcasses still needs symbiont supplementation for the formation of the adult cuticle. Concordantly, omnivorous *Camponotus* ants utilize endosymbionts that nutritionally upgrade their diet by supplementing tyrosine, and it is speculated that the symbiont acquisition was a major evolutionary step for this group of ants [83]. Similarly, it is conceivable that this tyrosine supplementation is also needed in some Dasytinae despite the presumably carnivorous larval diet. Besides its relevance for the cuticle, tyrosine and its derivatives are also required for various other functions in insects, such as the biosynthesis of neurotransmitters and immune reactions, but likely in lower quantities [84]. Furthermore, there might be additional functions of tyrosine precursors in *Dasytastes*-clade symbionts. The intermediate chorismate can also be used as a precursor for folate (vitamin B9) biosynthesis, and additional amino acids and vitamins are likely supplied by the symbiont to its host.

Tyrosine-provisioning symbionts have been reported for several beetles and ants, and their contribution to host fitness is based on enhanced desiccation resistance and protection against predators and pathogens [11–16]. The presence of tyrosine-providing symbionts in Dasytinae beetles indicates that tyrosine is a limiting resource across many taxa with diverse feeding habits, and the acquisition of nutritional endosymbionts is a widespread strategy to compensate for this deficiency.

### Symbiont functions beyond tyrosine provisioning

*Dasytastes*-clade strains retain a more diverse metabolic portfolio including several amino acid and vitamin biosynthesis pathways, as well as a urease. Thus, in addition to tyrosine and phenylalanine, *Dasytastes*-clade strains can synthesize the essential amino acids histidine, lysine, methionine, and threonine (Fig. 5, Fig. S11), deficiencies of which can severely lower insect fitness [85]. This hints at additional needs of the *Dasytastes*-clade hosts, which therefore exhibit species-specific selective pressures on their respective symbionts. Most likely, selection for the maintenance of these biosynthetic pathways weakened in non-*Dasytastes*-clade strains and thus they were rapidly lost, as the biosynthesis of some of these amino acids is especially costly [86]. Comparable patterns are found in many symbiotic systems, such as in Donaciinae (Chrysomelidae) beetles, whose symbionts retain genes encoding pectinolytic enzymes only in host species that feed on pectin-rich plants [23].

The presence of a urease suggests that symbiont-harboring beetles from the *Dasytastes*-clade are confronted with a particularly nitrogen-deficient diet. Urea is an abundant waste product of insect nitrogen metabolism, and urea recycling symbionts have been described in several insect taxa [87]. For example, the *Blattabacterium* endosymbiont of cockroaches recycles host-derived urea and uses it as a nitrogen source for amino acid biosynthesis [88], and urease genes have recently been described for the symbionts of silvanid and bostrichid beetles [13, 14], suggesting a role in nitrogenous waste recycling in beetles feeding on wood or stored grain products. Similarly, the urease-catalyzed hydrolysis of urea by *Dasytastes*-clade symbionts may provide ammonia that the bacterium could use to synthesize glutamine, aspartate, and glutamate, important precursors for the biosynthesis of several amino acids. Alternatively, the initially synthesized glutamine might be transported directly to the host and fuel its amino acid metabolism.

Beyond essential amino acid biosynthesis and nitrogen recycling, B vitamins are another group of compounds that most insects cannot synthesize [89], yet they are pivotal for development, adult survival, and reproduction [90, 91]. Many insects feeding on B-vitamin-deficient diets rely on endosymbionts that provide these compounds. Examples include not only the blood-feeding tsetse flies [92] and bedbugs [93] but also the seed-feeding cotton stainer *Dysdercus fasciatus* [94]. Symbionts of *Dasytastes*-clade hosts can synthesize and potentially supplement riboflavin (B2), pyridoxine (B6), and folate (B9) to their hosts (Fig. 5, Fig. S11). Riboflavin can be crucial for insects during development as well as for adult survival [91]. Furthermore, it is a precursor of flavin mononucleotide (FMN) and flavin adenine dinucleotide (FAD), important cofactors for flavoproteins [95]. Even though the enzymatic steps for FMN and FAD synthesis are missing in *Dasytiphilus*, they can be performed by a plethora of organisms [96]. Vitamin B6 is a relevant cofactor for many enzymes in insects [91]. One of its vitamers, pyridoxal 5′-phosphate (PLP), is involved in the conversion of DOPA into dopamine [97], an essential step for cuticle sclerotization and melanization. Furthermore, PLP was shown to be involved in amino acid metabolism, cofactor biosynthesis, and cell wall metabolism, among other processes, in *Bacillus subtilis* [98]. Folate is involved in the metabolism of amino acids and nucleic acids and can be crucial during insect development [91].

In addition to nutritional deficiencies in the diet, many herbivorous insects are confronted with the challenge of breaking down the plant cell wall. Palynivorous (i.e. pollen-feeding) species can utilize several mechanisms to overcome the recalcitrant pollen wall and gain access to the nutritious content [35]. Additionally, cell wall polymers can be degraded using digestive enzymes provided by bacterial symbionts [23, 99]. Based on the pollen-feeding habit of many adult Dasytinae and the close proximity of the bacteriomes to the gut, we previously speculated that *Dasytiphilus* may contribute to the break-down of the pollen [36]. However, the genomic analysis of *Dasytiphilus* genomes did not reveal any genes encoding for plant cell wall–degrading enzymes, so it seems unlikely that the symbionts contribute to the breakdown of pollen. Moreover, based on our detection of pollen in guts of Dasytinae adults from species with and without *Dasytiphilus* (Fig. S8), these beetles seem to be able to access pollen nutrients without the help of *Dasytiphilus*. It is possible that Melyridae beetles access the pollen content by using osmotic shock and inducing pseudogermination or by enlisting the help of gut bacteria that supply pectin-degrading enzymes, strategies that are also used by bees [100]. Alternatively, they might encode pectinolytic enzymes in their own genomes to break open the pollen [101].

### Differences in cell envelope biosynthesis between *Dasytiphilus* strains

Our genomic analysis revealed that several symbiont strains lost the ability to produce certain cell envelope components (Fig. 5), i.e. peptidoglycan and cardiolipin, a lipid that is used in the inner membrane. There are multiple potential explanations for these losses, which indicate that these deficient strains have further adapted to their life in the host environment. A complex cell envelope might not be essential anymore if the hosts provide a suitable environment. It is known that the intracellular environments of endosymbionts are often isotonic with the symbiont cytoplasm, diminishing the importance of a cell wall for maintaining turgor pressure [102]. Alternatively, hosts sometimes contribute to their endosymbionts’ cell wall formation with the help of horizontally acquired genes [25, 103]. These genes can be highly expressed in bacteriocytes harboring the endosymbiont, strongly suggesting a supportive role in, but also active control over symbiont cell envelope construction [104]. Even though uncommon, it is not possible to rule out similar mechanisms in the Dasytinae symbiosis without information on the host genomes.

### *Dasytiphilus* genome evolution

Similar to other nutritional endosymbionts, *Dasytiphilus* strains exhibit eroded genomes of very small size with low GC content. The largest changes in the symbiont genomes likely occurred in the early stages of the symbiotic association. This is reflected in the high degree of genomic similarity and synteny between clades as well as a small number of inversions of parts of the symbiont genomes between clades (Fig. 3). Genome size, gene content, and order within each clade are almost perfectly conserved (Fig. 5, Figs S4 and S10), providing additional evidence that most of the genome erosion happened early in the history of the symbiosis. However, the current genomes are still subject to ongoing erosion, which is likely facilitated by the reduced repertoire of DNA repair genes (Table S4). An example that might foreshadow the evolutionary trajectory of the *Dasytiphilus* genomes in closely related symbiont strains is the lysine biosynthesis pathway in *Listrus*-clade symbionts. The different states of this pathway could indicate reduced selective pressures on some *Listrus* symbionts for the supplementation of lysine. Interestingly, the dN/dS analysis revealed that in the *Listrus*-clade strains that retained the lysine pathway genes, these genes were still under purifying selection, albeit less strongly than in *Dasytastes* strains. Therefore, the loss of these genes in these *Listrus* spp. symbionts seems unlikely, indicating that loss patterns of certain capabilities alone do not necessarily predict future gene losses in closely related strains.

### Bacteriome structure and symbiont transmission

Like many nutritional endosymbionts in Hemiptera, Coleoptera, Diptera, and Hymenoptera, *Dasytiphilus* is localized in specialized bacteriomes. The bacteriomes of Dasytinae are located in very close proximity to the gut. In multiple Dasytinae species, accessory organs of the Malpighian tubules have been described that are touching the bacteriomes and are connected to the gut [81, 105]. However, these accessory organs and thus the connections from the bacteriome to the gut are absent in Dasytinae adult males [81]. This sex-specific difference raises the possibility of a vertical transmission route via the gut and the surface on the eggs, as is observed for symbionts located in the gut or gut-associated organs of many Heteroptera and some Coleoptera [106, 107]. In accordance with an extracellular transmission route via the gut, we never observed *Dasytiphilus* in the ovaries by FISH (Fig. S6); however, one *D. plumbeus* individual contained bacteria labeled with the *Dasytiphilus*-specific probe in the gut (Fig. S7). A potential behavioral indicator for transmission via the gut from the egg surface has been described for young larvae of Dasytinae beetles, which burst open the egg and then stay motionless in the egg for a few days [31, 33]. By doing so, they may be acquiring the symbionts from the egg surface and allowing time for the colonization of the bacteriomes via the gut. This feature is unique to Dasytinae beetles and differs from the behavior of beetles from other Melyridae subfamilies and the closely related Rhadalidae [108]. Concordantly, mature *P*. *viridicoerulea* larvae of both sexes have a direct connection from the gut to the bacteriome [81]. This untested hypothesis of the symbiont transmission route is an intriguing scenario, as it would—to our knowledge—provide the only known example of a transmission route that involves a phase outside the insect body for a bacteriome-localized symbiont. If true, the Dasytinae symbiosis might offer unprecedented insights into the evolutionary origin of bacteriomes. To test our hypothesized transmission route, future investigations should include the localization of the symbiont during the egg stage. Furthermore, manipulations during this life stage, like surface sterilization of the eggs, could lead to aposymbiotic larvae/adults, experimentally testing for an extracellular phase of the symbionts.

## Conclusion

By investigating the taxonomic distribution, tissue localization, and putative function of the *Dasytiphilus* symbiont within Dasytinae beetles, we elucidated the evolutionary history and dynamics of this symbiosis. Following a single acquisition event, four distinct host clades retained *Dasytiphilus* and show clear signs of host–symbiont coevolution, whereas multiple symbiont losses occurred in independent host lineages. The bacteriome-localized symbiont contains an eroded genome, and its main contribution to the host’s fitness likely resides in the supplementation of tyrosine that supports cuticle sclerotization and melanization. However, the symbionts of some host taxa probably also complement the host’s diet by producing additional essential amino acids and vitamins, as well as by recycling urea. The host ecological traits correlating with the differences in symbiont-provided benefits or the complete losses of symbionts remain unknown, due to the scarcity of knowledge on the natural history and feeding ecology of Dasytinae. The close proximity of the Dasytinae bacteriomes to the digestive tract, the occasional presence of the symbionts in the gut, and their absence from the ovaries raises the intriguing possibility of an extracellular transmission route of the bacteriome-localized symbiont. As such, the Dasytinae symbiosis provides an interesting system to study the evolutionary origin of bacteriome-localized symbionts as well as the ecological correlates of changes in symbiont presence and function, and it extends our understanding of tyrosine supplementation as an important symbiont-provided benefit across a broad range of insect taxa with diverse nutritional ecologies.

## Supplementary Material

Wierz_et_al_supplement_14_05_2024_wrae080

Supplementary_file_02-cog_kegg_wrae080

Supplementary_file_03-dnds_wrae080

## Data Availability

The datasets presented in this study are deposited in online repositories. The BioProject PRJNA1068458 on NCBI contains the Metagenome-Assembled Genomes (MAG) for all 20 assembled *Dasytiphilus* strains, as well as the underlying raw sequences in an SRA database (SRP485589). In addition, the annotated symbiont genomes are available in Edmond, which is a research data repository of the Max Planck Society (https://doi.org/10.17617/3.PFIWPC).
