# Supplementary material for: Evolutionary history of tyrosine-supplementing endosymbionts in pollen-feeding beetles
Source: ISME J. 2024 Jun 11;18(1):wrae080. doi: 10.1093/ismejo/wrae080 (PMC11191362; doi:10.1093/ismejo/wrae080)
Supplement: Wierz_et_al_supplement_14_05_2024_wrae080 [file wierz_et_al_supplement_14_05_2024_wrae080.pdf]

Supplementary material

Evolutionary history of tyrosine supplementing endosymbionts in pollen-feeding beetles

Jürgen C. Wierz[1,2], Matthew L. Gimmel[3], Selina Huthmacher[2], Tobias Engl[1,2], Martin Kaltenpoth[1,2*]

[1] Department of Insect Symbiosis, Max Planck Institute for Chemical Ecology, 07745 Jena, Germany
[2] Department of Evolutionary Ecology, Institute of Organismic and Molecular Evolution, Johannes Gutenberg University, 55128 Mainz, Germany
[3] Department of Invertebrate Zoology, Santa Barbara Museum of Natural History, Santa Barbara, CA 93105, USA
* Corresponding author: Martin Kaltenpoth, Department of Insect Symbiosis, Max Planck Institute for Chemical Ecology, Hans-Knöll-Str. 8, D-07745 Jena Germany, kaltenpoth@ice.mpg.de

Supplementary results

Amino acids and B-vitamin biosynthesis pathways in the symbiont genomes

Differences in symbiont genome lengths were also reflected in their metabolic capability (Figure 5); however, some capabilities were universally retained. All *Dasytiphilus* strains retained glycolysis and pentose phosphate pathways, both utilizing ß-D-Fructose-6P as the starting metabolite. However, the citrate cycle was incomplete, as the symbionts only encode genes for the enzymatic steps from 2-oxoglutarate to oxaloacetate via succinate. Furthermore, all *Dasytiphilus* encoded genes for the complete tyrosine and phenylalanine biosynthetic pathways (*aroB*, *aroQ*, *aroE*, *aroK*/*L*, *aroA*, *aroC*, *aroF*/*G*/*H*, *tyrA* and *aspC*). For the final steps in these pathways, aspartate aminotransferase *aspC* was encoded, which can take over the enzymatic activity usually performed by the more common aromatic-amino-acid transaminase encoded by tyrB (1–3). However, these final steps to tyrosine and phenylalanine can also be taken over by the host (4–6). Furthermore, *Dasytastes*-clade and *Listrus*-clade symbionts encoded *pheA* in addition to *tyrA* for the reaction from prephenate to phenylpyruvate. *Listrus* sp. 07 was missing the *aroQ* gene; however, it is likely that this gene absence was an artefact resulting from the low assembly quality of this specific symbiont's draft-genome consisting of 90 contigs with a coverage below 10. All *Dasytastes*-clade symbionts and several *Listrus*-clade symbionts had incomplete but likely functional lysine biosynthesis diaminopimelate pathways. All of these symbionts lacked the *argD*/*dapC* gene. Additionally, the *Listrus*-clade symbionts lacked the *lysA* gene. Furthermore, the *dapE* gene was absent in *Listrus* sp. 00 and *Listrus* sp. 09, and it was pseudogenized in *Listrus* sp. 02, *Listrus* sp. 04, and *Listrus* sp. 07. Lastly, the pathway was completely lost in the symbiont of *Listrus* sp. 01 as well as *Dasytes*-clade symbionts, and non-functional in the symbiont of *Listrus* sp. 06 and *Danacea*-clade symbionts, where we found heavily pseudogenized gene remnants. The genes encoding for *dapC*/*argD* are commonly missing in closely related endosymbionts, e.g. in some *Buchnera*, *Blochmania* and *Sodalis* endosymbionts. It is therefore possible that this specific catalytic reaction in the lysine biosynthesis pathway is taken over by other enzymes. It was shown in *Escherichia coli* that phosphoserine aminotransferase (*serC*) can perform this step (7). Interestingly, the *serC* gene was present in all analyzed *Dasytastes* and *Listrus* symbionts which otherwise had a nearly complete lysine synthesis pathway, whereas it was missing in *Danacea* and *Dasytes* symbionts that lacked the complete lysine pathway. This indicates that the *serC* gene indeed takes over the *dapC*/*argD* gene function in the lysine pathway in *Dasytiphilus.* The *dapE* gene is also known to be lost from several other endosymbionts with reduced genomes, e.g. *Tremblaya* symbiont of mealybugs or *Sulcia* symbionts (8,9). Remnants

of the gene were present in various *Listrus*-clade symbionts but appeared to be pseudogenized. A direct candidate gene that might take over this enzymatic step could not be identified, but it is possible that this function is taken over by another aminotransferase gene. Moreover, for the sometimes missing *lysA* gene, there are indications that the *speA* gene can take over this enzymatic step at lower efficiency (10). However, this *speA* gene was present in all *Dasytastes*-clade symbionts, but absent in *Listrus*-clade symbionts, and only the latter were missing the *lysA* gene. An alternative completion to this pathway might be done by the host. Many insects, e.g. mealybugs and whiteflies, encode *lysA* orthologs in their genomes (8,11). Another option is that the last step is not necessary because the symbiont only synthesizes lysine precursors as cell-envelope components. A similar scenario was hypothesized by Andersson et al. (12), but it seems unlikely in this case, as the very closely related *Dasytastes* symbionts still encoded for *lysA*. Symbionts from the *Dasytastes*-clade also encoded genes for the pathways of the essential amino acids histidine, methionine, and threonine.

Furthermore, *Dasytastes*-clade symbionts encoded genes for incomplete but probably functional biosynthetic pathways for the B-vitamins Riboflavin (B2), Pyridoxine (B6), and Folate (B9). The enzymatic pathway for riboflavin is branched and requires one molecule of guanosine 5'-triphosphate (GTP) and two molecules of ribulose 5-phosphate. *Dasytastes*-clade symbionts encode genes for all necessary enzymes. There is some uncertainty about the enzymatic step that catalyzes the dephosphorylation of 5-amino-6-(5'-phospho-D-ribitylamino)uracil to 5-amino-6-(1-D-ribitylamino)uracil. This step is done by an enzyme that was elusive for a long time and only recently several candidates were published (13–15), all belonging to the haloacid dehalogenase (HAD) superfamily. The detected candidate genes are quite different and the similarity between coding sequences of these putative genes is low (15). *Dasytastes*-clade symbionts encoded the yigL gene, which was also found in *Serratia* endosymbiont of aphids, where it was hypothesized to encode an enzyme that catalyzes the missing enzymatic step (16). Therefore, we speculate that the yigL gene fulfills the same function in *Dasytiphilus* and that this pathway is fully functional.

Additionally, symbionts of the *Dasytastes*-clade are likely able to synthesize Vitamin B6, which refers to a group of vitamers (pyridoxine (PN), pyridoxal (PL), pyridoxamine (PM)), and their respective 5′-phosphorylated forms (pyridoxine 5′-phosphate (PNP), pyridoxal 5′-phosphate (PLP), and pyridoxamine 5′-phosphate (PMP)) (17,18). The symbionts carry the genes necessary to synthesize phosphorylated B6 vitamers from D-erythrose-4-phosphate and also for dephosphorylating PLP. The other dephosphorylated forms could probably be acquired with the

help of unspecific phosphatases. Interestingly, the *dxs* gene was missing, which encodes an enzyme in a branching pathway to synthesize 1-deoxy-D-xylulose 5-phosphate (DXP). However, this metabolite can also be synthesized with the help of DXP reductoisomerase, an enzyme encoded by the *dxr* gene (19), which was present in the symbionts.

Moreover, symbionts of the *Dasytastes*-clade, with the exception of *Dasytes* sp. 02, were presumably able to synthesize the vitamin folate (B9). They encoded all but two genes needed for the branched biosynthesis pathway that requires guanosine 5'-triphosphate (GTP) and chorismate. The missing enzymatic steps would be performed by alkaline phosphatase and dihydrofolate reductase. However, it was shown that the gene encoding alkaline phosphatase might to not get annotated well, and an alleged lack in genome annotations cannot necessarily be used to rule out a complete folate biosynthesis pathway (20). Furthermore, this particular function might also be taken over by other multifunctional phosphatases. The other enzyme, dihydrofolate reductase, is usually encoded by the gene *folA*. The genomes of several strains of *Dasytiphilus* across all four clades were annotated to carry this gene, including *Dasytes* sp.01 and *Dasytes* sp.02. Due to the high gene synteny in *Dasytiphilus* strains, we were able to find the *folA* gene in all symbionts. Even though several mutations occurred, protein blast identified the putative gene sequence as *folA*, thus we hypothesize this gene to be functional in all symbionts.

Other putative symbiont functions

Besides biosynthetic pathways encoding metabolites that are potentially provided to the host, further differences between the symbiont strains existed. Symbionts from the *Dasytastes*-clade encoded genes for urease (encoded by the genes *ureA*, *ureB*, and *ureC*) and auxiliary proteins (encoded by the genes *ureD*/*ureH*, *ureE*, *ureF* and *ureG*) that catalyzes the hydrolysis of urea (21). The symbionts were lacking the *ureE* gene, which encodes a metallochaperone that binds nickel and can deliver it to the apoprotein (22), where it is important for the maturation of the urease. However, it was shown that *ureE* is not fundamental for this process and the apoprotein can obtain the nickel and become functional in *ureE*-deleted mutants, albeit with lower efficiency (22,23). It is therefore conceivable that the *Dasytastes*-clade symbionts encode for a functional urease. The urease-catalyzed hydrolysis of urea provides ammonia that the bacterium could use to synthesize glutamine with the help of glutamine synthetase encoded by the *glnA* gene. Subsequently, an enzyme complex encoded by *carA* and *carB* genes could use the glutamine to synthetize carbamoyl phosphate, a metabolite which functions as a precursor to aspartate and cytidine triphosphate (CTP) synthesis in the *Dasytastes*-clade symbionts. The latter pathway also

yields glutamate. Alternatively, the *carA* and *carB* enzyme complex can utilize excess ammonia directly to synthesize carbamoyl phosphate if glutamate and therefore glutamine is limited (24).

Some differences in the capability to synthesize metabolites important for the cell envelope existed. Whereas symbiont strains of the *Dasytastes*-clade could use the substrates glycerone phosphate or phosphatidylethanolamine to produce cardiolipin, all other symbionts only kept a gene for cardiolipin synthase to carry out the last enzymatic step. Additionally, symbiont strains in *P. viridicoerulea*, *D. aeratus*, *D. plumbeus* and *D. virens* lost the pathway to synthetize peptidoglycan.

Supplementary figures

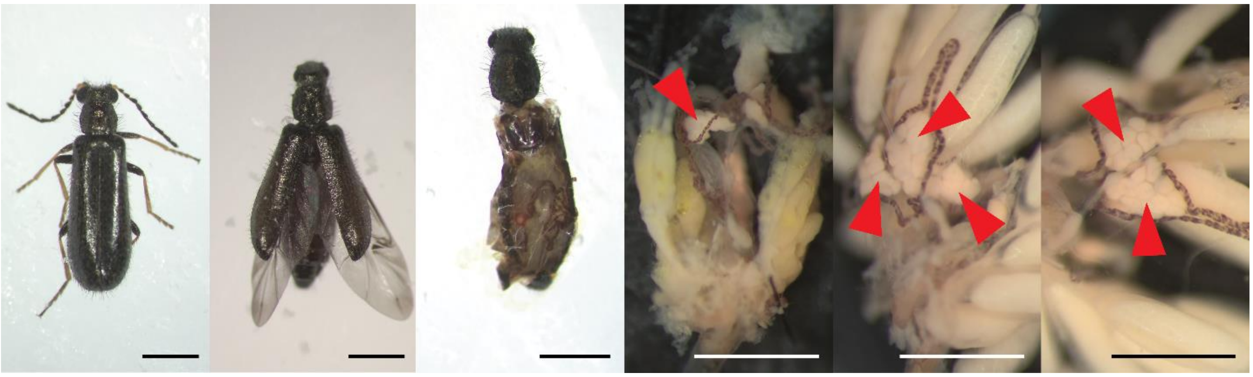

Figure S1:
Image series on dissection of bacteriomes from an adult *Dasytes plumbeus*. Red arrows show bacteriome, bar = 1 mm.

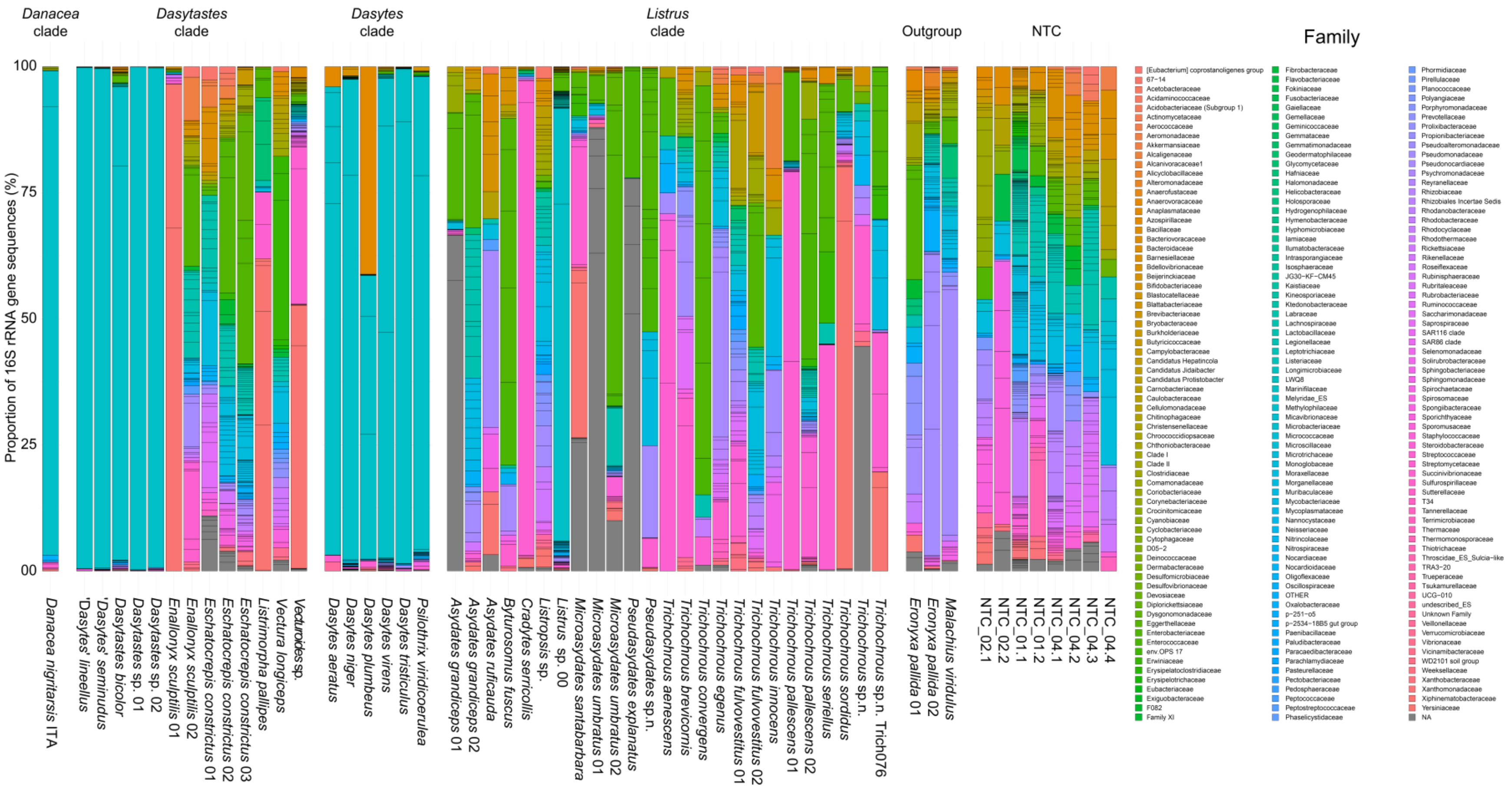

Figure S2: Bacterial community composition in Melyridae beetles given in relative abundance of bacterial amplicon sequence variants (ASVs) determined at family level by DADA2 analysis of Illumina 16S rRNA gene amplicons. Samples with less than 1,000 reads after removal of reads assigned to chloroplasts and mitochondria were excluded. Every bar represents a single individual, with DNA extracted from the whole body. The 2,000 most abundant bacterial ASVs are displayed with annotated family, remaining ASVs are grouped as "Other." NTC = No template extraction controls.

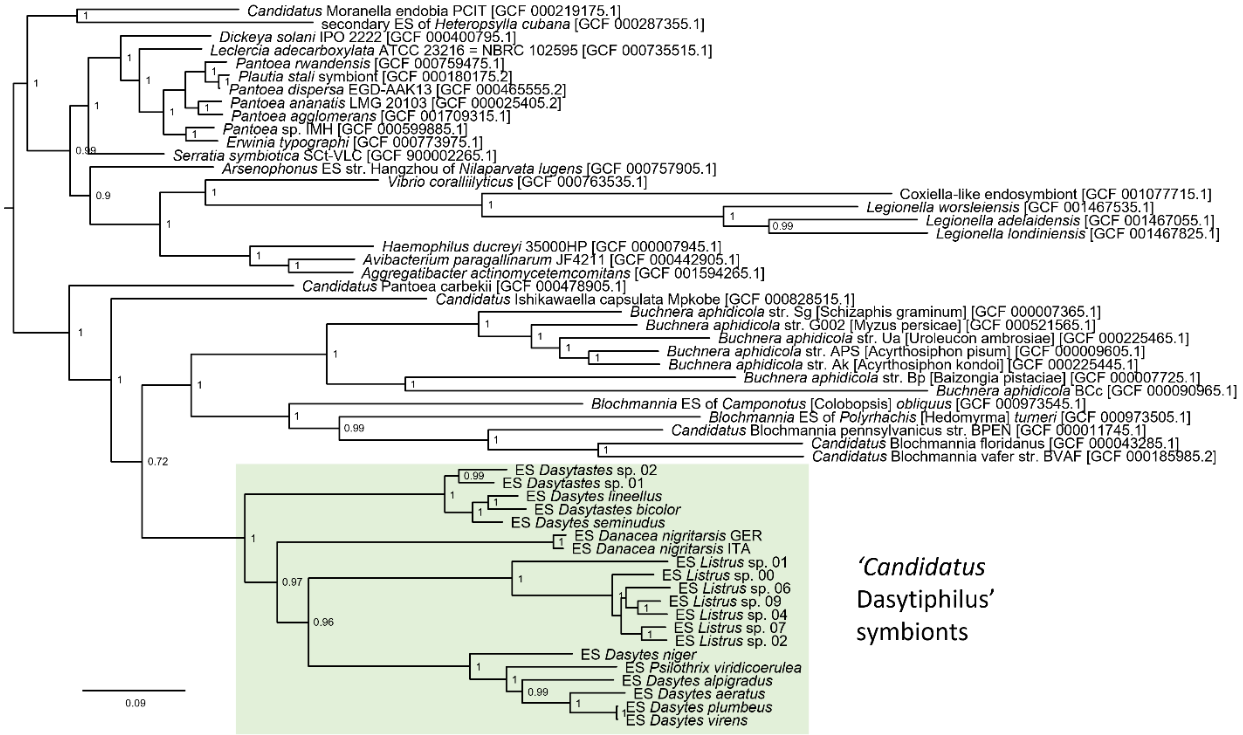

Figure S3:

Phylogenetic reconstruction of the different *Dasytiphilus* strains based on a set of 49 COG and done by using an approximately-maximum-likelihood algorithm. Node labels indicate local support values, with values below 70 removed.

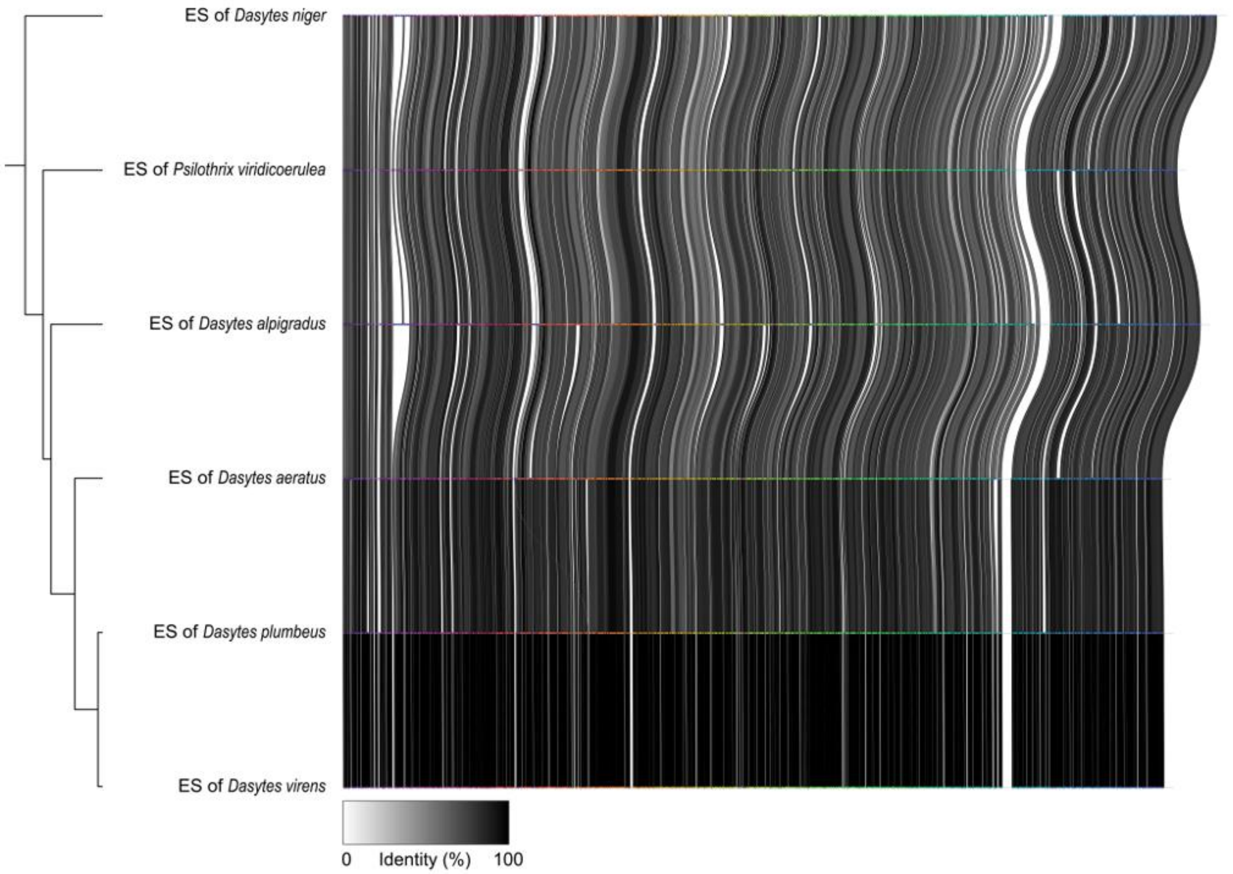

Figure S4: Genome synteny plot, comparing the gene order between the genomes of *Dasytiphilus* symbionts of hosts in the *Dasytes*-clade. The gene identity percentage of homologous proteins is based on amino acid sequences and indicated by differential grey values. The phylogeny on the left is based on a set of 49 COG and was reconstructed using an approximately-maximum-likelihood algorithm.

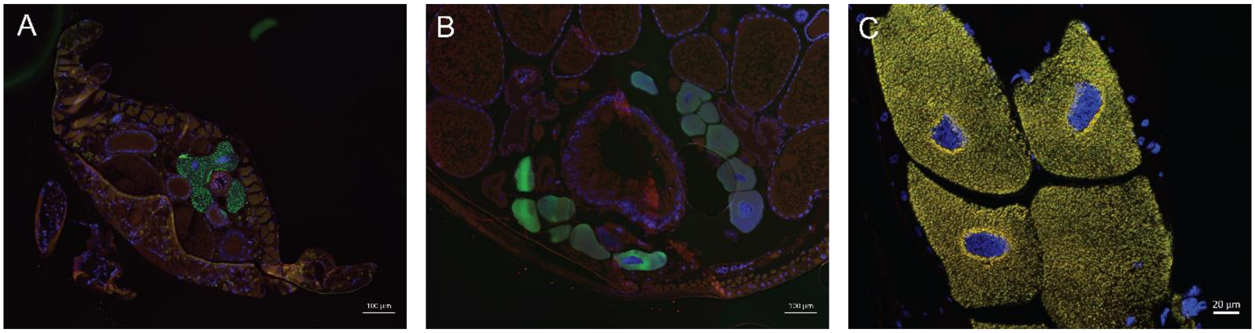

Figure S5:
Tissue localization of *Dasytiphilus* symbionts via fluorescence *in situ* hybridization in adult *Dasytes niger* (A), *Psilothrix viridicoerulea* (B), and *Dolichosoma lineare* (C). *Dasytiphilus* (in green or yellow) are aggregated in the bacteriocytes. No general bacteria (in red) are visible. Cell nuclei stained in blue with DAPI.

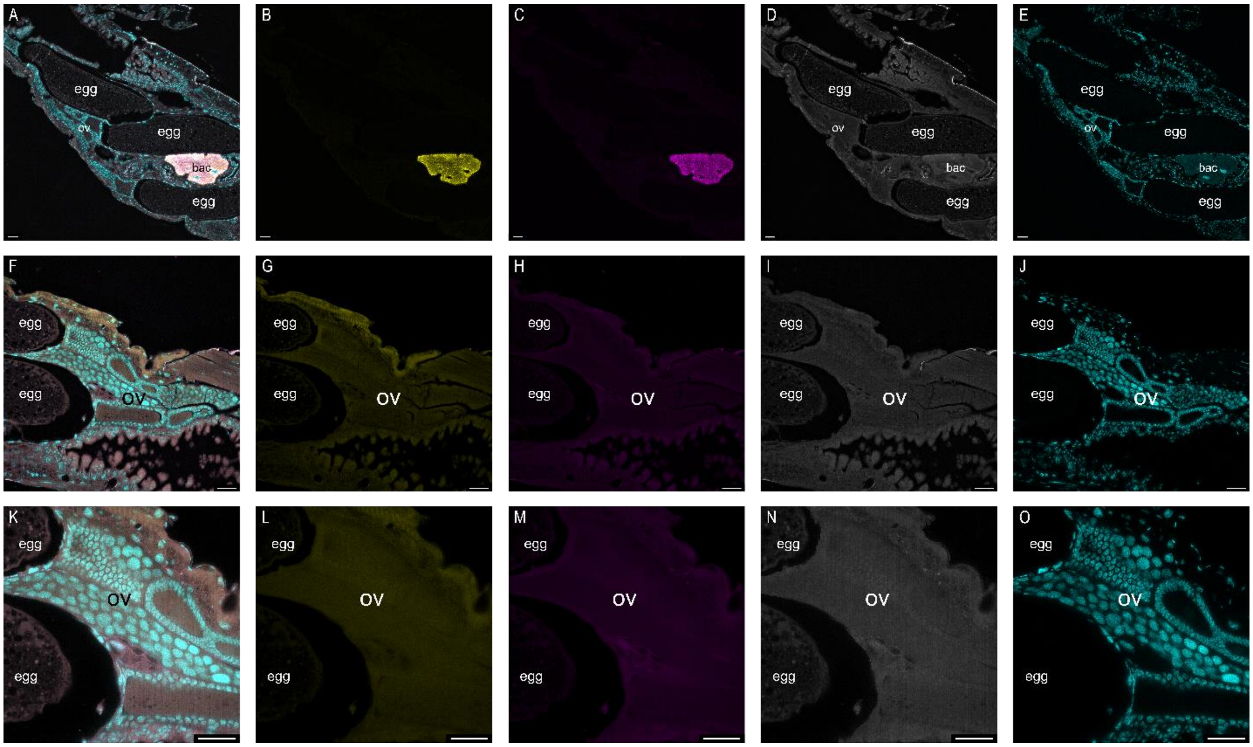

Figure S6:
Sagittal sections of the abdomen of *Dasytes plumbeus.* Using fluorescence *in situ* hybridization, *Dasytiphilus* symbionts were labeled specifically in magenta and non-specifically with a eubacterial probe in yellow. Background autofluorescence is given in white, and a general DNA counterstain in cyan (DAPI). Pictures A, F, and K show the overlaps of all four channels. Symbiont filled bacteriome (bac) is located towards the posterior of the abdomen (A-E). No bacterial signal was found in the ovaries (F-O) or in eggs that were still in the ovaries (ov). Scale bars = 50 µm.

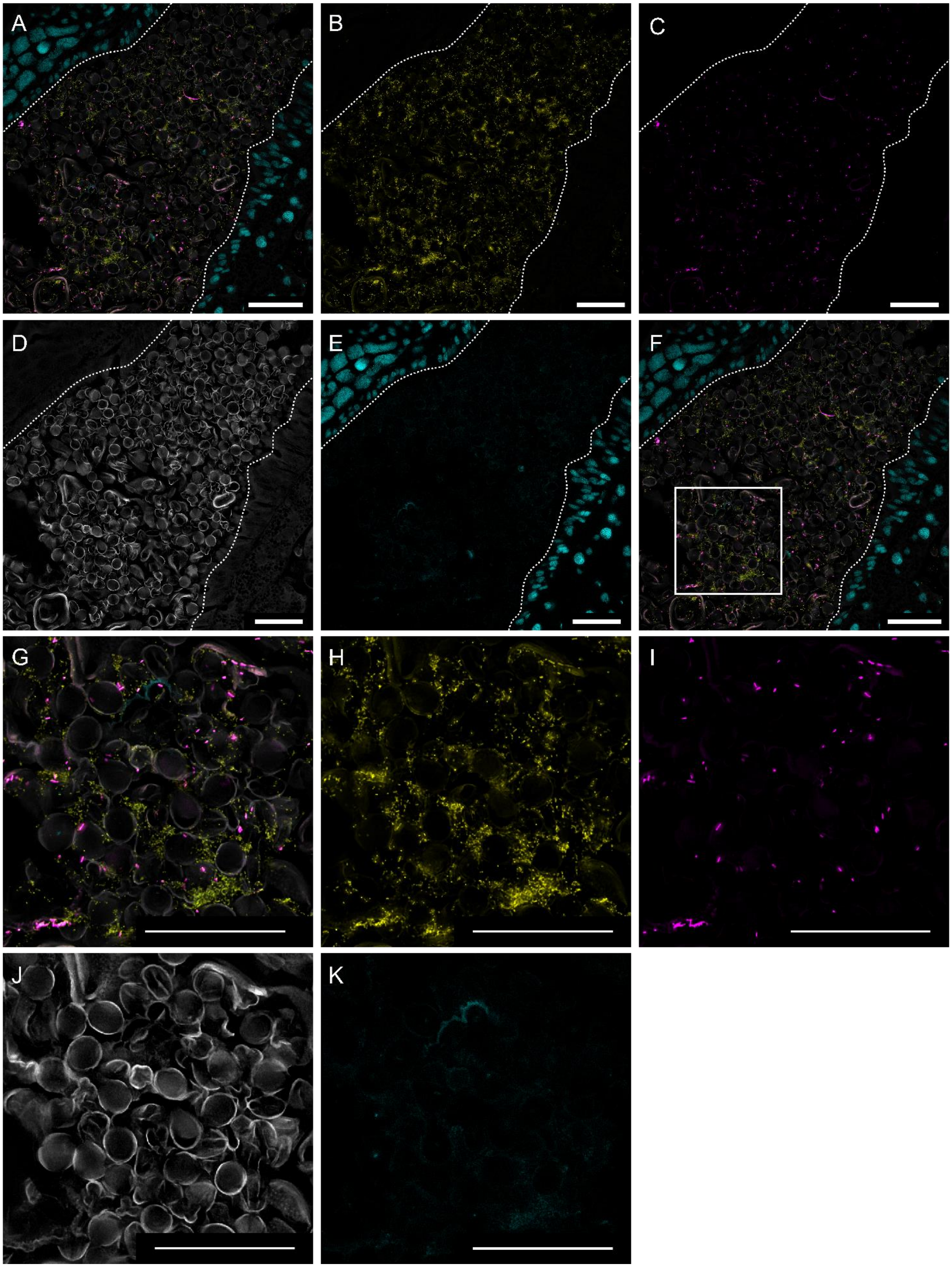
A
B
C
D
E
F
G
H
I
J
K

Figure S7:
Dasytiphilus symbionts were localized in the gut of an adult *Dasytes plumbeus* via fluorescence *in situ* hybridization of sagittal sections. The different panels show an overlay of all channels (A, F, G), eubacterial staining in yellow (B, H), *Dasytiphilus* specific staining in magenta (C, I), control staining is in white (D, J), and cell nuclei staining with DAPI in turquoise (E, K). White square in panel F shows the area depicted at higher magnification in panels G-K, with the channels corresponding to A-E. Scale bars = 50 µm. In between the visible consumed pollen grains many eubacterial cells can be seen, some of which are also stained by the *Dasytiphilus* specific probe. The shape of the *Dasytiphilus* cells in the gut lumen was slimmer and more elongated compared to the *Dasytiphilus* cells kept intracellularly within the bacteriome (see Figure 4), potentially as a result of the different environment.

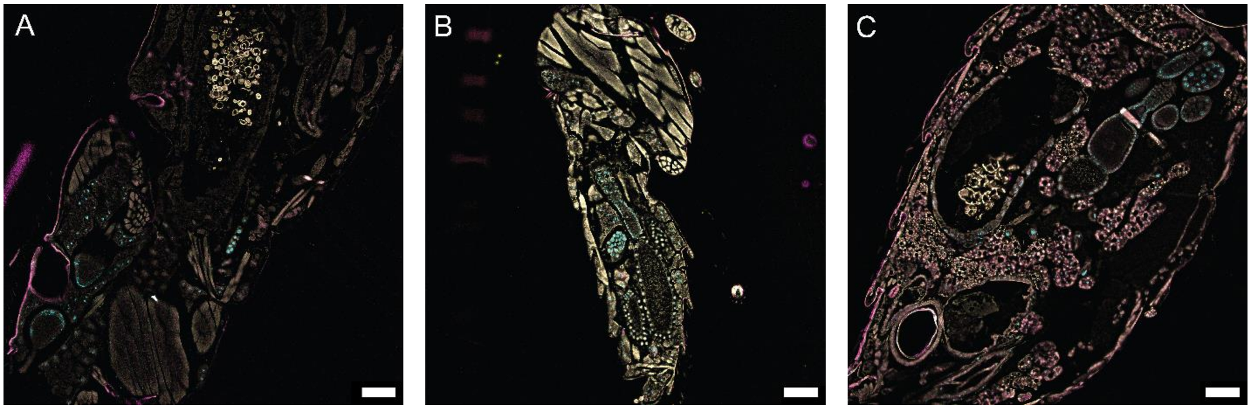

Figure S8:
No *Dasytiphilus* symbionts were found via fluorescence *in situ* hybridization in adult *Eschatocrepis constrictus* (A), *Gracilivectura pygidialis* (B), and *Trichochrous pallescens* (C). Control staining is in white and cell nuclei stained in turquoise with DAPI. Pictures shown are overlaps of all four channels. Consumed pollen grains were visible in the gut in all species. Scale bars = 100 µm.

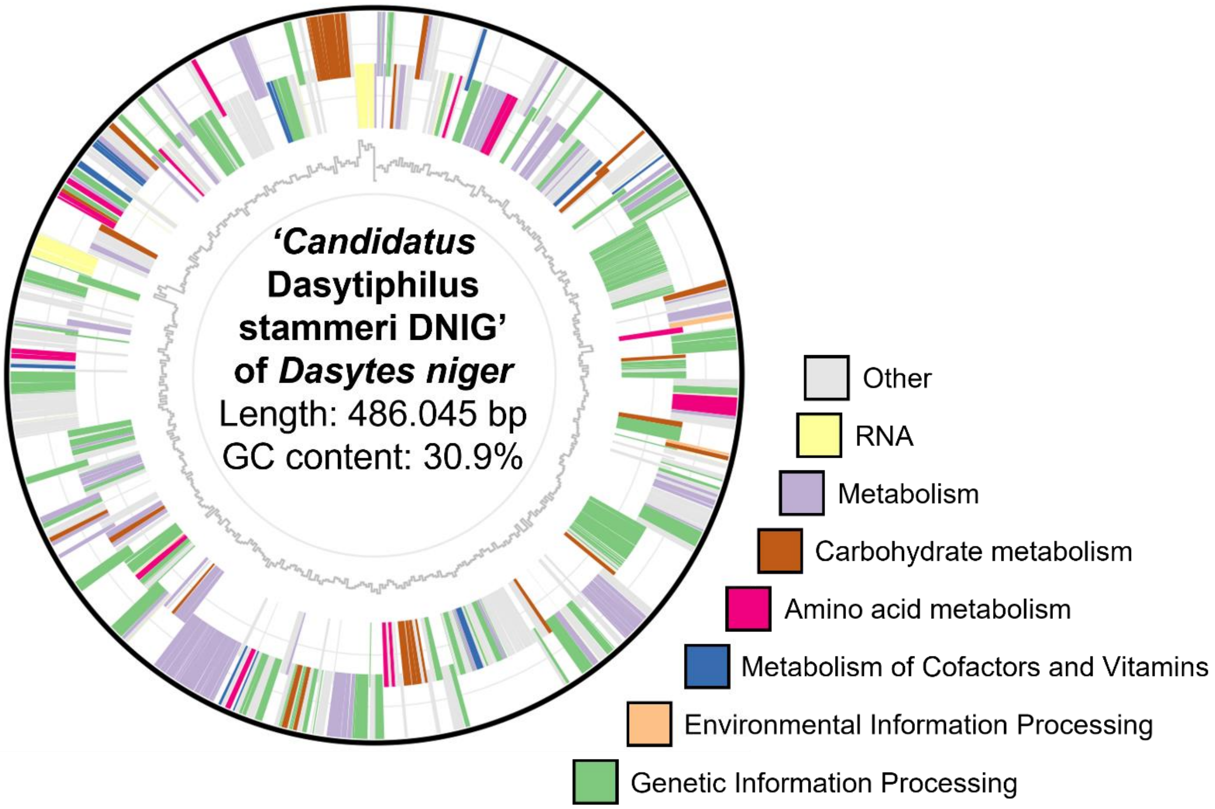

Figure S9: Representation of the circular genome of the *Dasytiphilus* symbiont of *Dasytes niger*. The inner gray line shows the relative GC content. Colored blocks depict genes separated on inner and outer circle based on direction of transcription, with color indicating the annotated functional KEGG categories.

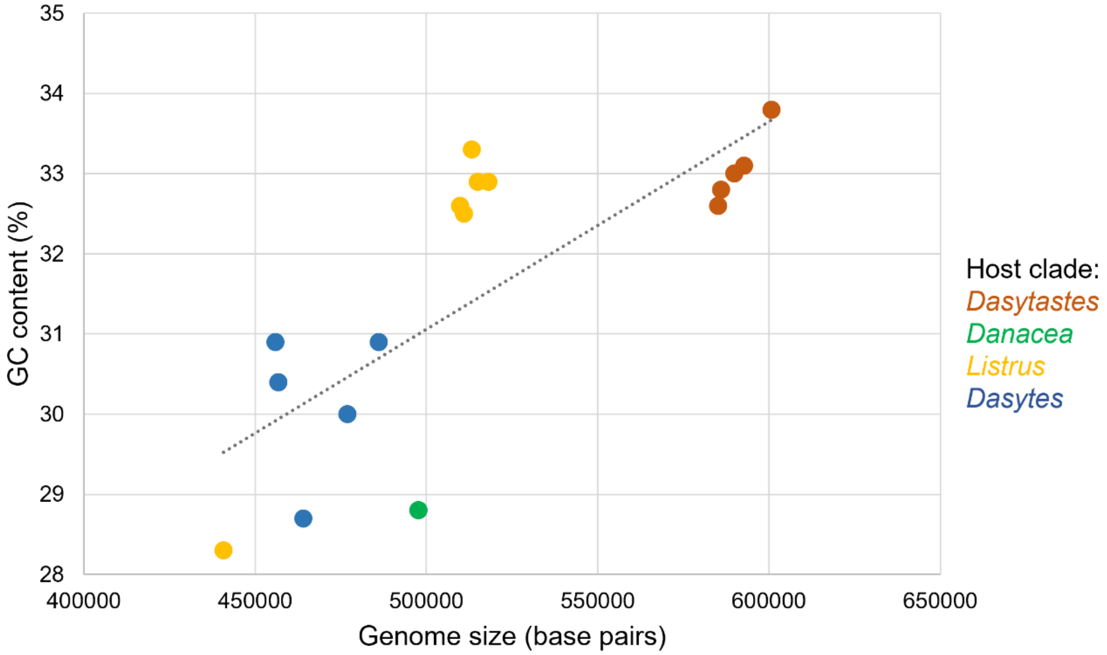

Figure S10: Correlation of genome length with GC content of *Dasytiphilus* symbionts. Dot colors indicate phylogenetic clade of host taxon for each symbiont strain. The dotted line represents the trendline.

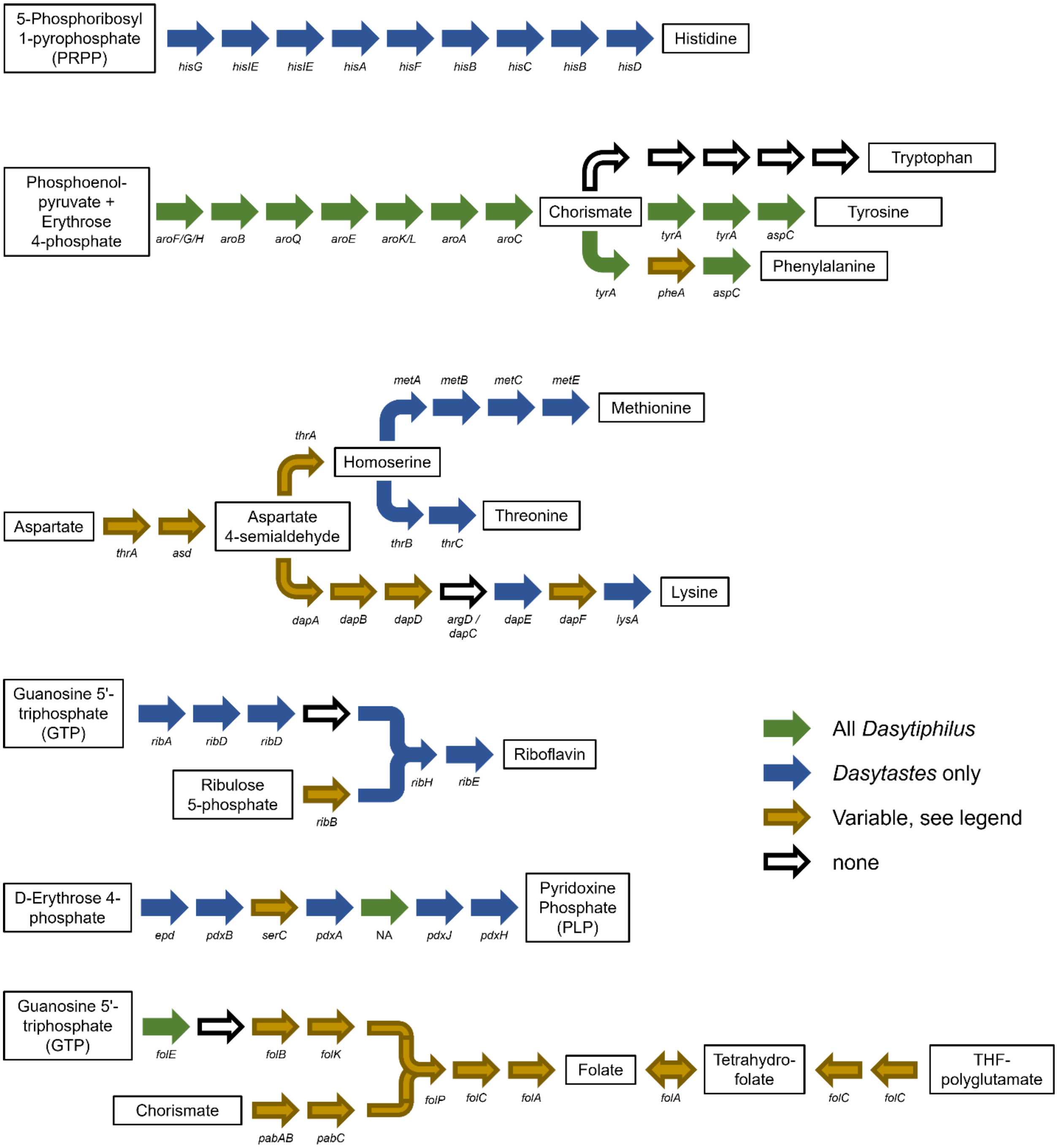

Figure S11:
Absence and presence of individual genes from selected amino acid and B-vitamin pathways. Each arrow stands for an enzymatic step, with the encoding gene given in italics. Green arrow indicate that this gene is present in all analyzed *Dasytiphilus* strains. Blue arrows indicate that this gene is present in all *Dasytiphilus* strains from the *Dasytastes*-clade, but absent in all other strains. Yellow arrows indicate a patchy distribution across *Dasytiphilus* strains, with explanations given below. Black and white arrow indicate that this gene is missing in all *Dasytiphilus* strains. Rectangular boxes represent key metabolites.
Phenylalanine pathway: gene *pheA* is only present in all *Dasytastes*-clade symbionts and all *Listrus*-clade symbionts, except in the endosymbiont (ES) of *Listrus* sp. 01.
Amino acid pathways starting from aspartate: Genes *thrA* and *asd* are only present in all *Dasytastes*-clade symbionts and all *Listrus*-clade symbionts, except in the ES of *Listrus* sp. 01. Genes *dapA*, *dapB*, *dapD*, and *dapF* are only present

in all *Dasytastes*-clade symbiont and all *Listrus*-clade symbionts, except in the ES of *Listrus* sp. 01 and *Listrus* sp. 06. However, pseudogenized remnants of the genes *dapB* and *dapF* are present in *Listrus* sp. 06.
Vitamin B2 Riboflavin pathway: The gene *serC* is only present in all *Dasytastes*-clade symbionts and all *Listrus*-clade symbionts, except in the ES of *Listrus* sp. 07, which could be a false-negative as a result of lower genome assembly quality.
Vitamin B6 Pyridoxine phosphate (PLP) pathway: The gene *serC* is only present in all *Dasytastes*-clade symbionts and all *Listrus*-clade symbionts, except in the ES of *Listrus* sp. 01. Also, this pathway includes a non-enzymatic step marked by NA.
Vitamin B9 Folate pathway: Genes *folB*, *folK*, *pabAB*, *pabC*, and *folP* are only present in all *Dasytastes*-clade symbionts, except in the ES of *Dasytastes* sp. 02. The gene *folC* is only present in all *Dasytastes*-clade symbionts except in the ES of *Dasytastes* sp. 02, and in all *Listrus*-clade symbionts, except in the ES of *Listrus* sp. 01. The gene *folA* is only present in the ES of *Dasytastes* sp. 01, *Dasytastes* sp. 02, *Danacea nigritarsis* GER, *Danacea nigritarsis* ITA, *Listrus* sp. 01, and all *Dasytes*-clade symbionts except in the ES of *Psilothrix viridicoerulea*.
In the biosynthetic pathways for the vitamins B1 (thiamine), B3 (Nicotinate), B5 (Pantothenate), B7 (Biotin), and B12 (Cobalamin), no genes were encoded in any of the *Dasytiphilus* strains, with the exception of the vitamin B1 (thiamine) pathway, in which the single gene *iscS* was present in all *Dasytiphilus* strains.

Supplementary tables

Table S1: Diagnostic and quantitative PCR primers, 16S microbial community amplicon primers and FISH probes used in this study.

Name	Sequence (5´--> 3´)	fwd./rev.	Label	Length	Target	Use	Reference
18S_ai	CCTGAGAAACGGCTA CCACATC	fwd	NA	22	18S	Host phylogeny	(25)
18S_a1.0	GGTGAAATTCTTGGA CCGTC	fwd	NA	20	18S	Host phylogeny	(25)
18S_3'I	CACCTACGGAAACCT TGTTACGAC	rev	NA	24	18S	Host phylogeny	(25)
28Sff	TTACACACTCCTTAG CGGAT	fwd	NA	20	28S	Host phylogeny	(26)
28Srr	GGGACCCGTCTTGAA ACAC	rev	NA	19	28S	Host phylogeny	(26)
C1-J-2183-F ("Jerry")	CAACATTTATTTTGAT TTTTTGG	fwd	NA	23	CO1	Host identification and phylogeny	(27)
TL2-N-3014-R ("Pat")	TCCAATGCACTAATC TGCCATATTA	rev	NA	25	CO1	Host identification and phylogeny	(27)
Melyridae CO1 F01	GGAGCWGTAAATTTT ATTACAAC	fwd	NA	23	CO1	Host identification and phylogeny	this study
Melyridae CO1 F02	ACAGTAATTAATATAC GWCC	fwd	NA	20	CO1	Host identification and phylogeny	this study
LepF1	ATTCAACCAATCATA AAGATATTGG	fwd	NA	25	CO1	Host identification and phylogeny	(28)
LepR1	TAAACTTCTGGATGT CCAAAAAATCA	rev	NA	26	CO1	Host identification and phylogeny	(28)
Sytb_F	TGAGGNCAAATATCH TTYTGAGG	fwd	NA	23	cytB	Host phylogeny	(29)
Sytb_R	GCAAATARRAARTAT CATTCDGG	rev	NA	22	cytB	Host phylogeny	(29)
fD1	AGAGTTTGATCCTGG CTCAG	fwd	NA	20	16S	Bacterial screening	(30)
rP2	ACGGCTACCTTGTTA CGACTT	rev	NA	21	16S	Bacterial screening	(30)
fD1_Mely	AGAGTTTGATCATGG CTCAG	fwd	NA	20	16S	Bacterial screening	this study
rP2_Mely	ACGGTTACCTTGTTA CGACTT	rev	NA	21	16S	Bacterial screening	this study
Dasy_Sym_fwd3	TAGAGGGAGATAGAA TTTCGGGTGT	fwd	NA	25	16S	*Dasytiphilus* specific screening	this study
Dasy_Sym_rev3	CCTTTGAGTTCCCGC CTTTA	rev	NA	20	16S	*Dasytiphilus* specific screening	this study
341f	CCTACGGGNGGCWG CAG	fwd	NA	17	16S	Microbial community amplicon V3-V4 forward	(31)
515F	GTGYCAGCMGCCGC GGTAA	fwd	NA	19	16S	Microbial community amplicon V4 forward	(32)
806bR	GGACTACNVGGGTW TCTAAT	rev	NA	20	16S	Microbial community amplicon V4 reverse	(33,34)
909R	CCGTCAATTCATTTG AGT	rev	NA	18	16S	Microbial community amplicon V4-V5 reverse	(35)
EUB338-Cy3	GCTGCCTCCCGTAG GAGT	rev	cy3	18	16S	FISH eubacteria staining	(36)
Dasy_ent_cy5	CCAATGGTTATCCCC CTCCA	rev	cy5	20	16S	FISH *Dasytiphilus* symbiont staining	(37)

Table S2: Regions of the 16S rRNA gene that were amplified during microbial community analysis for each sample.

Host species	Region	Clade
Danacea nigritarsis ITA	V4-V5	*Danacea*
'*Dasytes*' *lineellus*	V3-V4	*Dasytastes*
'*Dasytes*' *seminudus*	V3-V4	*Dasytastes*
Dasytastes bicolor	V4	*Dasytastes*
Dasytastes sp. 01	V3-V4	*Dasytastes*
Dasytastes sp. 02	V3-V4	*Dasytastes*
Enallonyx sculptilis 01	V3-V4	*Dasytastes*
Enallonyx sculptilis 02	V4	*Dasytastes*
Eschatocrepis constrictus 01	V3-V4	*Dasytastes*
Eschatocrepis constrictus 02	V4	*Dasytastes*
Eschatocrepis constrictus 03	V4	*Dasytastes*
Listrimorpha pallipes	V3-V4	*Dasytastes*
Vectura longiceps	V3-V4	*Dasytastes*
Vecturoides sp.	V4	*Dasytastes*
Dasytes aeratus	V4	*Dasytes*
Dasytes niger	V4	*Dasytes*
Dasytes plumbeus	V4	*Dasytes*
Dasytes tristiculus	V4-V5	*Dasytes*
Dasytes virens	V4	*Dasytes*
Psilothrix viridicoerulea	V4	*Dasytes*
Asydates grandiceps 01	V3-V4	*Listrus*
Asydates grandiceps 02	V4	*Listrus*
Asydates ruficauda	V3-V4	*Listrus*
Byturosomus fuscus	V3-V4	*Listrus*
Cradytes serricollis	V3-V4	*Listrus*
Listropsis sp.	V3-V4	*Listrus*
Listrus sp. 00	V4	*Listrus*
Microasydates santabarbara	V3-V4	*Listrus*
Microasydates umbratus 01	V3-V4	*Listrus*
Microasydates umbratus 02	V4	*Listrus*
Pseudasydates explanatus	V3-V4	*Listrus*
Pseudasydates sp.n.	V3-V4	*Listrus*
Trichochrous aenescens	V3-V4	*Listrus*
Trichochrous brevicornis	V3-V4	*Listrus*
Trichochrous convergens	V3-V4	*Listrus*
Trichochrous egenus	V3-V4	*Listrus*
Trichochrous fulvotarsis	V3-V4	*Listrus*
Trichochrous fulvovestitus 01	V3-V4	*Listrus*
Trichochrous fulvovestitus 02	V4	*Listrus*
Trichochrous pallescens 01	V3-V4	*Listrus*
Trichochrous pallescens 02	V4	*Listrus*
Trichochrous seriellus	V3-V4	*Listrus*
Trichochrous sordidus	V4	*Listrus*
Trichochrous sp.n.	V3-V4	*Listrus*
Trichochrous sp.n. Trich076	V3-V4	*Listrus*
Eronyxa pallida 01	V4	Outgroup
Eronyxa pallida 02	V4	Outgroup
Malachius viridulus	V4	Outgroup
NTC_01.1	V4	NA
NTC_01.2	V4	NA
NTC_02.1	V4-V5	NA
NTC_02.2	V4-V5	NA
NTC_03.1	V3-V4	NA
NTC_03.2	V3-V4	NA
NTC_03.3	V3-V4	NA
NTC_03.4	V3-V4	NA

Table S3: Sequencing systems and services used for metagenome sequencing of individual taxa.

Host genus	Host species	Clade	Sequencing system	Sequencing service
‘Dasytes’	*seminudus*	Dasytastes	HiSeq 3000	Max-Planck-Genome-center
‘Dasytes’	*lineellus*	Dasytastes	HiSeq 3000	Max-Planck-Genome-center
Dasytates	*bicolor*	Dasytastes	NovaSeq 6000	CeGaT GmbH
Dasytastes	sp.01	Dasytastes	HiSeq 3000	Max-Planck-Genome-center
Dasytastes	sp.02	Dasytastes	HiSeq 3000	Max-Planck-Genome-center
Danacea	*nigritarsis* ITA	Danacea	HiSeq 3000	Max-Planck-Genome-center
Danacea	*nigritarsis* GER	Danacea	NextSeq 2000	Max-Planck-Genome-center
Listrus	sp. 00	Listrus	NovaSeq 6000	CeGaT GmbH
Listrus	sp. 01	Listrus	NextSeq 2000	Max-Planck-Genome-center
Listrus	sp. 02	Listrus	NextSeq 2000	Max-Planck-Genome-center
Listrus	sp. 04	Listrus	NextSeq 2000	Max-Planck-Genome-center
Listrus	sp. 06	Listrus	NextSeq 2000	Max-Planck-Genome-center
Listrus	sp. 07	Listrus	NextSeq 2000	Max-Planck-Genome-center
Listrus	sp. 09	Listrus	NextSeq 2000	Max-Planck-Genome-center
Dasytes	*niger*	Dasytes	NovaSeq 6000	CeGaT GmbH
Psilothrix	*viridicoerulea*	Dasytes	NovaSeq 6000	CeGaT GmbH
Dasytes	*alpigradus*	Dasytes	NextSeq 2000	Max-Planck-Genome-center
Dasytes	*aeratus*	Dasytes	NextSeq 2000	Max-Planck-Genome-center
Dasytes	*plumbeus*	Dasytes	NovaSeq 6000	CeGaT GmbH
Dasytes	*virens*	Dasytes	HiSeq 3000	Max-Planck-Genome-center

Table S4: Overview of genes with DNA repair function and their presence in *Dasytiphilus* symbionts.

Gene	KO	Function	Presence
alkA	K01247	Base excision repair	none
Dam	K06223	Mismatch repair	none
dnaT	K02317	Homologous recombination	none
exoI	K01141	Mismatch repair	none
exoX / exoVII	K10857	Mismatch repair	none
Lig	K01972	DNA replication	all symbionts
Mfd	K03723	Nucleotide excision repair	none
Mpg	K03652	Base excision repair	none
mutH	K03573	Mismatch repair	all symbionts
mutL	K03572	Mismatch repair	all symbionts
mutM / fgp	K10563	Base excision repair	all symbionts
mutS	K03555	Mismatch repair	all symbionts
mutY	K03575	Base excision repair	none
nfo	K01151	Base excision repair	all symbionts
nth	K10773	Base excision repair	only *Danacea*-clade
priA	K04066	Homologous recombination	all symbionts
priB	K02686	Homologous recombination	only *Danacea*-clade
priC	K04067	Homologous recombination	none
recA	K03553	Homologous recombination	all symbionts
recB	K03582	Homologous recombination	all symbionts
recC	K03583	Homologous recombination	all symbionts
recD	K03581	Homologous recombination	none
recF	K03629	Homologous recombination	none
recG	K03655	Homologous recombination	none
recJ	K07462	Diverse DNA repair	none
recO	K03584	Homologous recombination	none
recR	K06187	Homologous recombination	none
ruvA	K03550	Homologous recombination	all symbionts
ruvB	K03551	Homologous recombination	all symbionts
ruvC	K01159	Homologous recombination	all symbionts
ssb	K03111	DNA replication	all symbionts
tag	K01246	Base excision repair	none
ung / udg	K03648	Base excision repair	In some*
uvrA	K03701	Nucleotide excision repair	none
uvrB	K03702	Nucleotide excision repair	none
uvrC	K03703	Nucleotide excision repair	none
uvrD / pcrA	K03657	Nucleotide excision repair	*Dasytastes*-clade, *Listrus*-clade (not Lis01)
radA	K04485	DNA repair and recombination proteins	*Dasytes*-clade
xseA	K03601	Mismatch repair	all symbionts
xseB	K03602	Mismatch repair	all symbionts

*Gene ung / udg was present in: *Danacea nigritarsis* GER, *Dasytastes bicolor*, *Dasytastes* sp. 01, *Dasytastes* sp. 02, *‚Dasytes‘ lineellus*, *‚Dasytes‘ seminudus*, *Listrus* sp. 00, *Listrus* sp. 02, *Listrus* sp. 04, *Listrus* sp. 06, *Listrus* sp. 09, *Dasytes niger*, *Dasytes alpigradus*, *Dasytes aeratus*, *Dasytes plumbeus*, *Dasytes virens*.

Table S5: Adaptation of Table 1, but giving the number of analyzed specimens for each individual analysis. A red number means that the respective analysis has shown that the symbiont was absent, green numbers represent symbiont presence. For analyses that only used a single specimen, always a female was used.

Host		Collection	Specimens used for each analysis				
Genus	Species	Country	Symbiont present	PCR	Sanger	FISH	Amplicon
Eronyxa	*pallida*	USA, CA	no	1	1		2
Malachius	*bipustulatus*	Germany	no			1	
Malachius	*viridulus*	USA, CA	no				1
Dasytastes	*bicolor*	USA, CA	yes	1			1
'Dasytes'	*lineellus*	USA, CA	yes	1	1		1
'Dasytes'	*seminudus*	USA, CA	yes	1	1		1
Dasytastes	sp. 01	USA, CA	yes	1	1		1
Dasytastes	sp. 02	USA, CA	yes	1	1		1
Enallonyx	*sculptilis*	USA, CA	no	2	2		2
Eschatocrepis	*constrictus*	USA, CA	no	2	2	1	3
Gracilivectura	*pygidialis*	USA, CA	no	1	1	1	1
Listrimorpha	*pallipes*	USA, CA	no	1	1		1
Vectura	*longiceps*	USA, CA	no	1	1		1
Vecturoides	sp.	USA, CA	no				1
Danacea	*nigritarsis* GER	Germany	yes	1	1	2	
Danacea	*nigritarsis* ITA	Italy	yes	1	1	1	1
Danacea	*pallipes*	Germany	yes	1	1		
Asydates	*grandiceps*	USA, CA	no	2	2		2
Asydates	*ruficauda*	USA, CA	no	1	1		1
Byturosomus	*fuscus*	USA, CA	no	1	1		1
Cradytes	*serricollis*	USA, NM	no	1	1		1
Cradytes	*serrulatus*	USA, AZ	no	1	1		1
Eudasytes	*grandicollis*	USA, NV	no	1	1		1
Listropsis	sp.	USA, CA	no	1	1		1
Listrus	sp. 00	USA, CA	yes	1	1		2
Listrus	sp. 01	USA, AZ	yes	1		2	
Listrus	sp. 02	USA, CA	yes	1			
Listrus	sp. 03	USA, NV	yes	1	1		
Listrus	sp. 04	USA, UT	yes	1			
Listrus	sp. 05	USA, CA	yes	1	1		
Listrus	sp. 06	USA, CA	yes	1			
Listrus	sp. 07	USA, CA	yes	1			
Listrus	sp. 08	USA, CA	yes	1	1		
Listrus	sp. 09	USA, OR	yes	1			
Listrus	sp. 10	USA, OR	yes	1	1	2	
Microasydates	*santabarbara*	USA, CA	no	1	1		1
Microasydates	*umbratus*	USA, CA	no	2	2		2
Pseudasydates	*explanatus*	USA, CA	no	1	1		1
Pseudasydates	sp.n.	USA, NM	no	1	1		1
Trichochrous	*aenescens*	USA, CA	no	1	1		1
Trichochrous	*brevicornis*	USA, CA	no	1	1		1
Trichochrous	*convergens*	USA, AZ	no	1	1		1
Trichochrous	sp.n.	USA, CA	no	1	1		1
Trichochrous	*egenus*	USA, CA	no	1	1		1
Trichochrous	*fulvotarsis*	USA, CA	no	1	1		1
Trichochrous	*fulvovestitus*	USA, CA	no	2	2		2
Trichochrous	*pallescens*	USA, CA	no	2	2	1	2
Trichochrous	*quadrinotatus*	USA, CA	no	1	1		1
Trichochrous	*seriellus*	USA, UT	no	1	1		1
Trichochrous	*sordidus*	USA, CA	no	2	2		2
Trichochrous	sp.n. Trich076	USA, CA	no	1	1		1
Dasytes	*aeratus*	Germany	yes	1	1		1
Dasytes	*alpigradus*	Italy	yes	1	1		
Dasytes	*niger*	Germany	yes	4	4	2	1

Dasytes	*plumbeus*	Germany	yes	10	10	6	1
Dasytes	*subaeneus*	Germany	yes	1	1		
Dasytes	*tristiculus*	Italy	yes	1	1	1	1
Dasytes	*virens*	Germany	yes	22	5	2	5
Dolichosoma	*lineare*	Germany	yes	1	1	2	
Psilothrix	*viridicoerulea*	Germany	yes	1	1	1	1

Supplementary references

1. Gelfand DH, Steinberg RA. *Escherichia coli* mutants deficient in the aspartate and aromatic amino acid aminotransferases. J Bacteriol. 1977 Apr;130(1):429–40.

2. Hayashi H, Inoue K, Nagata T, Kuramitsu S, Kagamiyama H. *Escherichia coli* aromatic amino acid aminotransferase: characterization and comparison with aspartate aminotransferase. Biochemistry. 1993;32(45):12229–39.

3. Powell JT, Morrison JF. The purification and properties of the aspartate aminotransferase and aromatic-amino-acid aminotransferase from *Escherichia coli*. Eur J Biochem. 1978 Jun;87(2):391–400.

4. Anbutsu H, Moriyama M, Nikoh N, Hosokawa T, Futahashi R, Tanahashi M, et al. Small genome symbiont underlies cuticle hardness in beetles. Proc Natl Acad Sci USA. 2017 Oct 3;114(40).

5. Klein A, Schrader L, Gil R, Manzano-Marín A, Flórez L, Wheeler D, et al. A novel intracellular mutualistic bacterium in the invasive ant *Cardiocondyla obscurior*. ISME J. 2016 Feb;10(2):376–88.

6. Wilson ACC, Ashton PD, Calevro F, Charles H, Colella S, Febvay G, et al. Genomic insight into the amino acid relations of the pea aphid, *Acyrthosiphon pisum*, with its symbiotic bacterium *Buchnera aphidicola*: amino acid metabolism genes in the pea aphid symbiosis. Insect Mol Biol. 2010 Feb 23;19:249–58.

7. Lal PB, Schneider BL, Vu K, Reitzer L. The redundant aminotransferases in lysine and arginine synthesis and the extent of aminotransferase redundancy in *Escherichia coli*: Aminotransferase redundancy. Mol Microbiol. 2014 Nov;94(4):843–56.

8. Husnik F, Nikoh N, Koga R, Ross L, Duncan RP, Fujie M, et al. Horizontal gene transfer from diverse bacteria to an insect genome enables a tripartite nested mealybug symbiosis. Cell. 2013 Jun;153(7):1567–78.

9. McCutcheon JP, Moran NA. Functional convergence in reduced genomes of bacterial symbionts spanning 200 My of evolution. Genome Biol Evol. 2010 Jan 1;2:708–18.

10. Liao S, Poonpairoj P, Ko KC, Takatuska Y, Yamaguchi Y, Abe N, et al. Occurrence of Agmatine Pathway for Putrescine Synthesis in *Selenomonas ruminatium*. Biosci Biotechnol Biochem. 2008 Feb 23;72(2):445–55.

11. Bao XY, Yan JY, Yao YL, Wang YB, Visendi P, Seal S, et al. Lysine provisioning by horizontally acquired genes promotes mutual dependence between whitefly and two intracellular symbionts. Jiggins FM, editor. PLoS Pathog. 2021 Nov 29;17(11):e1010120.

12. Andersson SGE, Zomorodipour A, Andersson JO, Sicheritz-Pontén T, Alsmark UCM, Podowski RM, et al. The genome sequence of *Rickettsia prowazekii* and the origin of mitochondria. Nature. 1998 Nov;396(6707):133–40.

13. Haase I, Sarge S, Illarionov B, Laudert D, Hohmann HP, Bacher A, et al. Enzymes from the haloacid dehalogenase (HAD) superfamily catalyse the elusive eephosphorylation step of riboflavin biosynthesis. ChemBioChem. 2013 Nov 25;14(17):2272–5.

14. Sa N, Rawat R, Thornburg C, Walker KD, Roje S. Identification and characterization of the missing phosphatase on the riboflavin biosynthesis pathway in *Arabidopsis thaliana*. Plant J. 2016 Dec;88(5):705–16.

15. Sarge S, Haase I, Illarionov B, Laudert D, Hohmann HP, Bacher A, et al. Catalysis of an essential step in vitamin B_2 biosynthesis by a consortium of broad spectrum hydrolases. ChemBioChem. 2015 Nov;16(17):2466–9.

16. Manzano-Marín A, Simon JC, Latorre A. Reinventing the wheel and making it round again: evolutionary convergence in *Buchnera* – *Serratia* symbiotic consortia between the distantly related Lachninae aphids *Tuberolachnus salignus* and *Cinara cedri*. Genome Biol Evol. 2016 May;8(5):1440–58.

17. Fitzpatrick TB, Amrhein N, Kappes B, Macheroux P, Tews I, Raschle T. Two independent routes of *de novo* vitamin B6 biosynthesis: not that different after all. Biochem J. 2007 Oct 1;407(1):1–13.

18. Rosenberg IH. A history of the isolation and identification of vitamin B_6. Ann Nutr Metab. 2012;61(3):236–8.

19. Takahashi S, Kuzuyama T, Watanabe H, Seto H. A 1-deoxy- D -xylulose 5-phosphate reductoisomerase catalyzing the formation of 2- *C* -methyl- D -erythritol 4-phosphate in an alternative nonmevalonate pathway for terpenoid biosynthesis. Proc Natl Acad Sci USA. 1998 Aug 18;95(17):9879–84.

20. Celik OF, O'Sullivan DJ. Correlation of gene content in selected bifidobacteria with folate supplier or scavenger status during growth in laboratory media. Food Biosci. 2023 Feb;51:102324.

21. Patra AK, Aschenbach JR. Ureases in the gastrointestinal tracts of ruminant and monogastric animals and their implication in urea-N/ammonia metabolism: A review. J Adv Res. 2018;13:39–50.

22. Lee MH, Pankratz HS, Wang S, Scott RA, Finnegan MG, Johnson MK, et al. Purification and characterization of *Klebsiella aerogenes* UreE protein: A nickel-binding protein that functions in urease metallocenter assembly. Protein Sci. 1993 Jun;2(6):1042–52.

23. Brayman TG, Hausinger RP. Purification, characterization, and functional analysis of a truncated *Klebsiella aerogenes* UreE urease accessory protein lacking the histidine-rich carboxyl terminus. J Bacteriol. 1996 Sep;178(18):5410–6.

24. Rubino SD, Nyunoya H, Lusty CJ. In vivo synthesis of carbamyl phosphate from NH3 by the large subunit of *Escherichia coli* carbamyl phosphate synthetase. J Biol Chem. 1987 Mar;262(9):4382–6.

25. Shull VL, Vogler AP, Baker MD, Maddison DR, Hammond PM. Sequence alignment of 18S ribosomal RNA and the basal relationships of adephagan beetles: evidence for monophyly

of aquatic families and the placement of Trachypachidae. Simon C, editor. Syst Biol. 2001 Nov 1;50(6):945–69.

26. Bocakova M, Constantin R, Bocak L. Molecular phylogenetics of the melyrid lineage (Coleoptera: Cleroidea). Cladistics. 2012;28(2):117–29.

27. Simon C, Frati F, Beckenbach A, Crespi B, Liu H, Flook P. Evolution, weighting, and phylogenetic utility of mitochondrial gene sequences and a compilation of conserved polymerase chain reaction primers. Ann Entomol Soc Am. 1994;87(6):651–701.

28. Hebert PDN, Penton EH, Burns JM, Janzen DH, Hallwachs W. Ten species in one: DNA barcoding reveals cryptic species in the neotropical skipper butterfly *Astraptes fulgerator*. Proc Natl Acad Sci USA. 2004;101(41):14812–7.

29. Gimmel ML, Bocakova M, Gunter NL, Leschen RAB. Comprehensive phylogeny of the Cleroidea (Coleoptera: Cucujiformia). Syst Entomol. 2019;44(3):527–58.

30. Weisburg WG, Barns SM, Pelletier DA, Lane DJ. 16S ribosomal DNA amplification for phylogenetic study. J Bacteriol. 1991;173(2):697–703.

31. Herlemann DP, Labrenz M, Jürgens K, Bertilsson S, Waniek JJ, Andersson AF. Transitions in bacterial communities along the 2000 km salinity gradient of the Baltic Sea. ISME J. 2011;5(10):1571–9.

32. Parada AE, Needham DM, Fuhrman JA. Every base matters: assessing small subunit rRNA primers for marine microbiomes with mock communities, time series and global field samples. Environmental microbiology. 2016;18(5):1403–14.

33. Caporaso JG, Lauber CL, Walters WA, Berg-Lyons D, Huntley J, Fierer N, et al. Ultra-high-throughput microbial community analysis on the Illumina HiSeq and MiSeq platforms. ISME J. 2012;6(8):1621–4.

34. Caporaso JG, Lauber CL, Walters WA, Berg-Lyons D, Lozupone CA, Turnbaugh PJ, et al. Global patterns of 16S rRNA diversity at a depth of millions of sequences per sample. Proc Natl Acad Sci USA. 2011;108 Suppl 1:4516–22.

35. Kato C, Li L, Tamaoka J, Horikoshi K. Molecular analyses of the sediment of the 11,000-m deep Mariana Trench. Extremophiles : life under extreme conditions. 1997;1(3):117–23.

36. Amann RI, Binder BJ, Olson RJ, Chisholm SW, Devereux R, Stahl DA. Combination of 16S rRNA-targeted oligonucleotide probes with flow cytometry for analyzing mixed microbial populations. Environ Microbiol. 1990;56(6):1919–25.

37. Weiss B, Kaltenpoth M. Bacteriome-localized intracellular symbionts in pollen-feeding beetles of the genus *Dasytes* (Coleoptera, Dasytidae). Front microbiol. 2016;7:1486.
